# Identification of Novel Ghanaian G8P[6] Human-Bovine Reassortant Rotavirus Strain by Next Generation Sequencing

**DOI:** 10.1371/journal.pone.0100699

**Published:** 2014-06-27

**Authors:** Francis E. Dennis, Yoshiki Fujii, Kei Haga, Susan Damanka, Belinda Lartey, Chantal A. Agbemabiese, Nobuo Ohta, George E. Armah, Kazuhiko Katayama

**Affiliations:** 1 Department of Environmental Parasitology, Tokyo Medical and Dental University, Tokyo, Japan; 2 Laboratory of Gastroenteritis Viruses, Department of Virology II, National Institute of Infectious Diseases, Musashi-murayama, Tokyo, Japan; 3 Department of Electron Microscopy and Histopathology, Noguchi Memorial Institute for Medical Research, College of Health Sciences, University of Ghana, Legon, Ghana; Kobe University, Japan

## Abstract

Group A rotaviruses (RVAs) are the most important etiological agent of acute gastroenteritis in children <5 years of age worldwide. The monovalent rotavirus vaccine Rotarix was introduced into the national Expanded Programme on Immunization (EPI) in Ghana in May 2012. However, there is a paucity of genetic and phylogenetic data on the complete genomes of human RVAs in circulation pre-vaccine introduction. The common bovine rotavirus VP7 genotype G8 has been sporadically detected in Ghanaian children, usually in combination with the VP4 genotype P[6]. To investigate the genomic constellations and phylogeny of RVA strains in circulation prior to vaccine introduction, the full genomes of two unusual G8P[6] strains, GH018-08 and GH019-08, detected during burden of disease surveillance, were characterized by Illumina MiSeq sequencing. The Ghanaian isolates, GH018-08 and GH019-08, exhibited the unusual, previously unreported genotype constellation G8-P[6]-I2-R2-C2-M2-A2-N2-T2-E2-H3. Phylogenetic analyses confirmed that 10 out of the 11 genes of GH018-08 and GH019-08 were identical/nearly identical, with significant variation detected only in their VP1 genes, and clearly established the occurrence of multiple independent interspecies transmission and reassortment events between co-circulating bovine/ovine/caprine rotaviruses and human DS-1-like RVA strains. These findings highlight the contribution of reassortment and interspecies transmission events to the high rotavirus diversity in this region of Africa, and justify the need for simultaneous monitoring of animal and human rotavirus strains.

## Introduction

Group A rotaviruses (RVAs) are the most important etiological agent of acute gastroenteritis in children <5 years of age worldwide, and are estimated to be responsible for most of the 453,000 rotavirus-associated deaths annually, of which the majority occur in countries of sub-Saharan Africa and South East Asia [1]. RVAs, members of the genus *Rotavirus* within the family *Reoviridae*, characteristically possess a genome consisting of 11 double-stranded RNA segments that encode 6 viral structural proteins (VP1-VP4, VP6 and VP7) and 6 non-structural proteins (NSP1-NSP6) [2]. The traditional binary classification system for RVAs is based on the characteristics of the two outer capsid proteins VP7 (glycoprotein, G-genotype), and VP4 (protease-sensitive protein, P-genotype) [3], [4]. Unfortunately, this classification system cannot comprehensively define molecular epidemiology and evolutionary relationships among circulating RVA strains. Recently, an extended RVA classification and nomenclature system has been established based on the assignment of genotypes to all 11 genome segments [3], [5]. The complete genotype descriptor Gx-P[x]-Ix-Rx-Cx-Mx-Ax-Nx-Tx-Ex-Hx (“x” indicating the genotype number) represents the VP7-VP4-VP6-VP1-VP2-VP3-NSP1-NSP2-NSP3-NSP4-NSP5- encoding gene segments, respectively. Currently, 27 G- and 37 P-genotypes have been identified [5], [6]. While at least 73 G/P genotype combinations have been described in humans [7], only a limited number of strains - G1P[8], G2P[4], G3P[8], G4P[8], G9P[8], and to a lesser extent G12P[8] and G12P[6] - are most frequently associated with the RVA disease burden globally [8]–[11].

Based on complete RVA genome sequence comparisons, three human genotype constellations of the non-G, non-P genes have been described: Wa-like (genotype 1), DS-1-like (genotype 2), and AU-1-like (genotype 3). This classification agrees with the RVA genogroups established by RNA-RNA hybridization assays, represented by prototype human RVA strains Wa, DS-1 and AU-1 [12]–[15].

The G8 genotype is common in bovine rotaviruses [16]–[20], and was previously only sporadically detected in humans, in whom zoonotic transmission was postulated [21]. Though globally uncommon in humans, the G8 genotype has been reported frequently among African diarrhoeic children, especially in Eastern, Central and Southern African countries, mainly in combination with the P[6] genotype [22]–[27]. This unusually high prevalence of G8 strains in Africa has been speculated to be the result of interspecies transmission of rotaviruses between humans and cattle [28]–[31]. However, in Ghana, G8 rotavirus strains have only been sporadically detected since the first strains were isolated in 1999 [32]–[36].

Ghana introduced the monovalent rotavirus vaccine Rotarix into the national Expanded Programme on Immunization (EPI) in May 2012, and has set in place a surveillance system to monitor rotavirus strains post-vaccine introduction, as well as the emergence of new strains. However, there is a paucity of genetic and phylogenetic data on the complete genomes of human RVA in circulation in Ghana; information that will allow the comparison of strains pre- and post-vaccine introduction. In this study, we describe the complete genome characterization of two unusual Ghanaian G8P[6] RVA strains.

## Materials and Methods

### Ethics Statement

This study involved the further characterization of stool samples collected as part of WHO rotavirus burden of disease surveillance studies in Ghanaian children carried out per the WHO generic protocol [37]. The study was approved by the Institutional Review Board, Noguchi Memorial Institute for Medical Research, Legon, Ghana. In the burden of disease study, written informed consent for the testing of stool samples for rotaviruses and characterization of identified rotavirus strains was obtained from children's parents/guardians prior to sample collection.

### Study samples

GH018-08 and GH019-08 were isolated in 2008 from children <5 years old with diarrhoea seeking medical attention at the Navrongo War Memorial Hospital in the Kassena-Nankana district of the Upper East Region of Ghana. They were part of 35 randomly selected, partially characterized, single infection RVA strains of various G- and P-genotype combinations from samples collected from the study site between 2008 and 2010. GH019-08 was associated with gastroenteritis severe enough to warrant hospitalization. GH018-08 and GH019-08 had been screened for RVA by Enzyme Immunoassay (EIA) using the commercial IDEIA enzyme immunoassay kit (Dako Diagnostics Ltd., Cambridgeshire, UK), and polyacrylamide gel electrophoresis (PAGE) of RNA as previously described [38]. VP7 and VP4 genotypes were determined by reverse transcription-polymerase chain reaction (RT-PCR) as described elsewhere [35]. Sample screening and RT-PCR based genotype characterization data for GH018-08 and GH019-08 are summarized in Table 1. Both GH018-08 and GH019-08 had sufficient bulk stool for further characterization.

**Table 1:** Summary of partial characterization data for GH018-08 and GH019-08.

Strain	Year	Age (months)	EIA	PAGE	E-type	Severity	Strain
GH018-08	2008	N/A	Neg	++++	L	N/A	G?P[6]
GH019-08	2008	8	Neg	+	L	H	G?P[6]

N/A: Not available; EIA: Enzyme Immunoassay; Neg: Negative; +: positive; L: Long electropherotype by PAGE; H: hospitalization as a measure of severity of rotavirus infection, G?: G-type not available

### RNA Extraction and building cDNA library

Viral RNA was extracted from 10∼20% faecal suspensions in PBS using the Direct-zol RNA MiniPrep kit (Zymo Research, Irvine, CA, USA) according to manufacturer's instructions. To assess the integrity of RVA dsRNA, polyacrylamide gel electrophoresis (PAGE) was carried out at constant current (30 mA per gel) on a precast 10% ePAGEL (ATTO Corporation, Tokyo, Japan). RVA dsRNA bands were visualized by SYBR Gold staining.

Using the Qubit RNA Assay, sample RNA concentrations were determined on a Qubit 2.0 Fluorometer (Invitrogen, Carlsbad, CA, USA), and starting concentrations normalized to 10∼100 ng for library construction. A 200 bp fragment library was constructed for each sample using the NEBNext Ultra RNA Library Prep Kit for Illumina v1.2 (New England Biolabs, Ipswich, MA, USA) according to manufacturer's instructions. Samples were bar-coded for multiplexing using NEBNext Multiplex Oligos for Illumina, Index Primer Sets 1 and 2 (New England Biolabs, Ipswich, MA, USA). Library purification was done using Agencourt AMPure XP magnetic beads (Beckman Coulter, Pasadena, CA, USA) as recommended in the NEBNext protocol. The quality of the purified libraries was assessed on a MultiNA MCE-202 bioanalyzer (Shimadzu Corporation, Kyoto, Japan) and the concentrations determined on a Qubit 2.0 flourometer using the Qubit HS DNA Assay (Invitrogen, Carlsbad, CA, USA).

### Nucleotide sequencing and sequence data analysis

A 151-cycle paired-end read sequencing run was carried out on a MiSeq desktop sequencer (Illumina, San Diego, CA, USA) using the MiSeq Reagent Kit v2 (300 cycles). Following preliminary analysis, the MiSeq reporter programme was used to generate FASTQ formatted sequence data for each sample. Sequence data was analyzed using CLC Genomics Workbench Software v6.5.1 (CLC Bio, Aarhus, Denmark). Contigs were assembled from obtained sequence reads by *de novo* assembly. Subsequently, assembled contig sequences were used to query the non-redundant nucleotide database in GenBank employing the Basic Local Alignment Search Tool [BLAST] (http://blast.ncbi.nlm.nih.gov/Blast.cgi). Genotypes of confirmed RVA sequences were then determined according to the recommendations of the Rotavirus Classification Working Group using the automated genotyping tool for Group A rotaviruses, RotaC v2.2b [3], [39]. Nucleotide sequences of successfully characterized RVA genes were aligned with cognate gene sequences available in GenBank using the ClustalX2.1 algorithm [40], and phylogenetic analyses were performed using the Neighbour-Joining method in MEGA v6.06 software [41]. Phylogenetic trees were statistically supported by bootstrapping with 2000 replicates, and phylogenetic distances calculated using the Kimura-2 parameter model.

### Nucleotide sequence accession numbers

The nucleotide sequence data for the complete genomes of strains GH018-08 (RVA/Human-wt/GHA/GH018-08/2008/G8P[6]) and GH019-08 (RVA/Human-wt/GHA/GH019-08/2008/G8P[6]) have been deposited in GenBank under the accession numbers KJ748465 – KJ748475 and KJ748476–KJ748486 respectively.

## Results

An assessment of the integrity of dsRNA genomes of the Ghanaian G8P[6] RVA strains GH018-08 and GH019-08 by PAGE revealed identical, unusual, long RNA migration patterns (Figure S1).

### Nucleotide sequencing and assignment of genotypes

Full-length or nearly full-length sequences of all 11 genomic segments of GH018-08 and GH019-08 were successfully determined by Illumina MiSeq sequencing. The lengths of nucleotide and deduced amino acid sequences of genomic segments for GH018-08 and GH019-08, with related sequence read data is summarized in Table S1. Of particular interest were the VP2 genes of GH018-08 (full length, 2690 bp) and GH019-08 (nearly full length, 2688 bp), which were longer than cognate genes of DS-1-like strains (artiodactyl, lapine, and equine: 2687 bp; human: 2684 bp) within GenBank. All the genome segments sequenced in this study belonged to already-established genotypes. GH018-08 and GH019-08 were assigned the unusual, previously unreported genotype constellation G8-P[6]-I2-R2-C2-M2-A2-N2-T2-E2-H3. The complete genotype assignments of GH018-08 and GH019-08, along with selected published reference RVA strains of known genomic constellation, are shown in Table 2. Sequences of cognate genes available in the non-redundant nucleotide database in GenBank were selected based on BLAST queries and included in phylogenetic analyses. The GenBank accession numbers for all included RVA strains are listed in Table S2.

**Table 2:** Genotype constellation of GH018-08 and GH019-08 with selected reference RVA strains of known genomic constellation.

Strain^a^	Genotype^b^										
	VP7	VP4	VP6	VP1	VP2	VP3	NSP1	NSP2	NSP3	NSP4	NSP5
**RVA/Human-wt/GHA/GH018-08/2008/G8P[6]**	G8†	P[6]	**I2**	**R2**	**C2**	**M2**	**A2**	**N2**	**T2**	**E2**	H3‡
**RVA/Human-wt/GHA/GH019-08/2008/G8P[6]**	G8†	P[6]	**I2**	**R2**	**C2**	**M2**	**A2**	**N2**	**T2**	**E2**	H3‡
RVA/Human-wt/BGD/Dhaka16/2003/G1P[8]	G1	P[8]	I1	R1	C1	M1	A1	N1	T1	E1	H1
RVA/Human-wt/CHN/TB-Chen/1996/G2P[4]	**G2**	**P[4]**	**I2**	**R2**	**C2**	**M2**	**A2**	**N2**	**T2**	**E2**	**H2**
RVA/Human-wt/USA/06-242/2006/G2P[6]	**G2**	P[6]	**I2**	**R2**	**C2**	**M2**	**A2**	**N2**	**T2**	**E2**	**H2**
RVA/Human-wt/BEL/F01322/2009/G3P[6]	G3	P[6]	**I2**	**R2**	**C2**	**M2**	**A2**	**N2**	**T2**	**E2**	**H2**
RVA/Human-tc/GBR/ST3/1975/G4P2A[6]	G4	P[6]	I1	R1	C1	M1	A1	N1	T1	E1	H1
RVA/Human-wt/HUN/Hun5/1997/G6P[14]	G6†	P[14]†	**I2**	**R2**	**C2**	**M2**	A11†	**N2**	T6†	**E2**	H3‡
RVA/Goat-tc/BGD/GO34/1999/G6P[1]	G6†	P[1]†	**I2**	**R2**	**C2**	**M2**	A11†	**N2**	T6†	**E2**	H3‡
RVA/Human-wt/BEL/B1711/2002/G6P[6]	G6†	P[6]	**I2**	**R2**	**C2**	**M2**	**A2**	**N2**	**T2**	**E2**	**H2**
RVA/Human-tc/IND/69M/1980/G8P[10]	G8†	P[10]†	**I2**	**R2**	**C2**	**M2**	**A2**	**N2**	**T2**	**E2**	**H2**
RVA/Human-wt/COD/DRC86/2003/G8P[6]	G8†	P[6]	**I2**	**R2**	**C2**	**M2**	**A2**	**N2**	**T2**	**E2**	**H2**
RVA/Human-wt/COD/DRC88/2003/G8P[8]	G8†	P[8]	**I2**	**R2**	**C2**	**M2**	**A2**	**N2**	**T2**	**E2**	**H2**
RVA/Sheep-tc/ESP/OVR762/2002/G8P[14]	G8†	P[14]†	**I2**	**R2**	**C2**	**M2**	A11†	**N2**	T6†	**E2**	H3‡
RVA/Human-wt/HUN/BP1062/2004/G8P[14]	G8†	P[14]†	**I2**	**R2**	**C2**	**M2**	A11†	**N2**	T6†	**E2**	H3‡
RVA/Human-wt/BEL/B3458/2003/G9P[8]	G9	P[8]	I1	R1	C1	M1	A1	N1	T1	E1	H1
RVA/Human-wt/ZAF/GR10924/1999/G9P[6]	G9	P[6]	**I2**	**R2**	**C2**	**M2**	**A2**	**N2**	**T2**	**E2**	**H2**
RVA/Human-wt/BEL/B4633/2003/G12P[8]	G12	P[8]	I1	R1	C1	M1	A1	N1	T1	E1	H1
RVA/Pigeon-tc/JPN/PO-13/1983/G18P[17]	G18	P[17]	I4	R4	C4	M4	A4	N4	T4	E4	H4

a RVA strain names according to proposed nomenclature of new classification system. **Boldface** font indicates RVA strains sequenced in this study. GenBank accession numbers for reference strains are listed in Table S2.

b DS-1-like genotypes indicated by **boldface** font;

† Typical bovine-like RVA genotypes.

‡ AU-1-like genotype.

### Phylogenetic Analyses

To further investigate the genetic relationships among GH018-08, GH019-08 and other RVA strains, phylogenetic analyses were conducted based on nucleotide sequences of the entire open reading frames of all 11 gene segments. Compared to commonly detected G2P[4] and G3P[6] Ghanaian RVA strains [33], as well as rare G4P[6] and G6P[6] genotypes bearing the DS-1-like genomic backbone I2-R2-C2-M2-A2-N2-T2-E2-H2, GH018-08 and GH019-08 possessed unique VP7 and NSP5 genotypes, and their VP2, VP3, VP6 and NSP2 genes clustered in unique lineages, while their VP4 (P[6] genotype), NSP1, NSP3 and NSP4 genes clustered with co-circulating DS-1-like Ghanaian strains (unpublished data). While the VP1 gene of GH018-08 formed a unique lineage separate from other DS-1-like Ghanaian strains within the R2 genotype, GH019-08 clustered in a separate sublineage within a major lineage (unpublished data).

**Figure 1:**
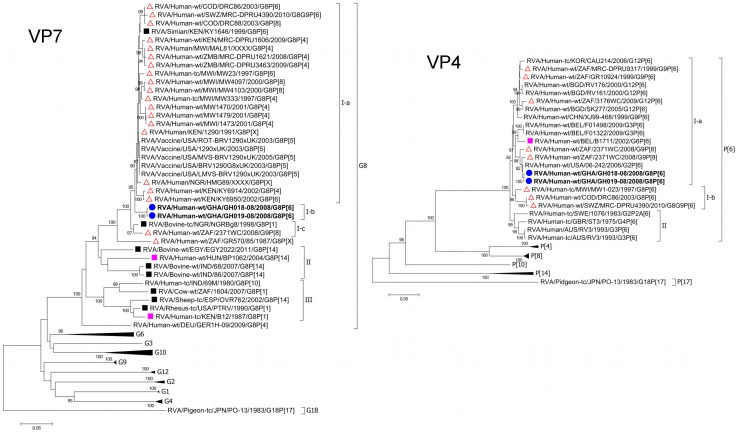
Phylograms based on the full-length open reading frame (ORF) nucleotide sequences of VP7 and VP4 encoding genome segments of RVA strains GH018-08 and GH019-08 indicating genetic relationships with cognate RVA genes. Neighbour-Joining trees were constructed and rooted with cognate genes of the avian RVA strain PO-13 using MEGA v6.06 software. Only the genotypes in which study strains cluster are shown completely. GH018-08 and GH019-08 are shown in boldface and indicated by a blue, closed circle, animal strains by a black closed box, reported human-artiodactyl reassortant strains by a closed pink box, multiple reassortant bovine-feline/canine-human strains by an open pink circle, and African human strains by an open red triangle. Significant bootstrap values (2000 replicates) of ≥80% are shown. Bar: nucleotide substitutions per site. GenBank Accession numbers for all strains are listed in Table S2.

The **VP7** genes of GH018-08 and GH019-08 were identical, forming a monophyletic cluster closely related to (95.3–97.7% nucleotide sequence identity), but distinct from human G8 strains, especially those of African origin (Figure 1, Tables S3, S4). Though the strain RVA/Human/1290/Kenya/1991/G8P[X] exhibited the highest nucleotide sequence identity (97.7%) to GH018-08 and GH019-08, the bovine strain RVA/Bovine-tc/NGR/NGRBg8/1998/G8P[1] [30] and the simian strain RVA/Simian/KEN/KY1646/1999/G8P[X] [26] were also quite closely related (96% and 97.3% nucleotide sequence identity respectively) (Figure 1, Table S4). KY1646 was collected from a vervet monkey experimentally infected with an unpassaged RVA strain obtained from the stool of a 6 year old child [42]. Human-artiodactyl reassortant strains, as well as several African and Indian bovine strains were distantly related and clustered in separate lineages (Figure 1).

The **VP4** genes of GH018-08 and GH019-08 were identical, and clustered closely with human P[6] genotypes, sharing 95.1–99.6% nucleotide sequence identity (Figure 1, Tables S3, S4). However, the African strains RVA/Human-tc/MWI/MW23/1997/G8P[6] [31], RVA/Human-wt/SWZ/MRC-DPRU4390/2010/G8G9P[6], and RVA/Human-wt/COD/DRC86/2003/G8P[6] [43] formed a separate sublineage. Though possessing similar nucleotide sequence identities as DRC86 to GH018-08 and GH019-08 (94.9%–95.3% and 95.8% respectively), rotavirus strains isolated before 1994- RVA/Human-tc/SWE/1076/1983/G2P2A[6], RVA/Human-tc/GBR/ST3/1975/G4P2A[6], and RVA/Human/AUS/RV3/1993/G3P[6] [12], [44]–[46]- clustered separately, forming a completely different lineage (Figure 1).

**Figure 2:**
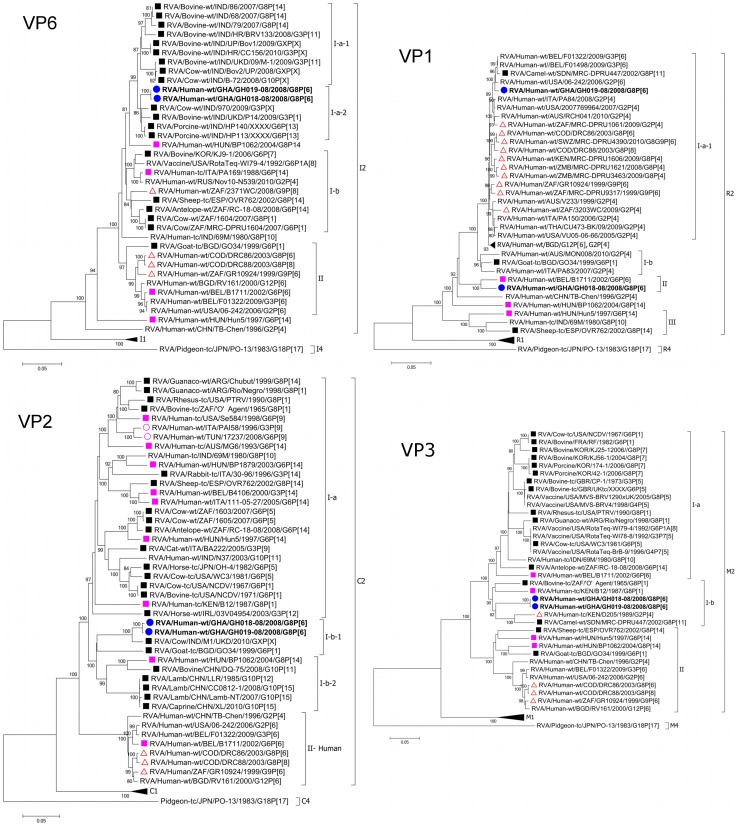
Phylograms based on the full-length open reading frame (ORF) nucleotide sequences of VP6, VP1, VP2 and VP3 encoding genome segments of RVA strains GH018-08 and GH019-08 indicating genetic relationships with cognate RVA genes. See Legend of Figure 1.

The **VP6** genes of GH018-08 and GH019-08 were identical, forming a monophyletic cluster within the I2 genotype. They were closely related (97.7–98.8% nucleotide sequence identity) to porcine (RVA/Porcine-wt/IND/HP113/XXXX/G6P[13] and RVA/Porcine-wt/IND/HP140/XXXX/G6P[13] [47]) and bovine (RVA/Cow-wt/IND/970/2009/G3P[X] and RVA/Bovine-wt/IND/UKD/P14/2009/G3P[1]) strains of Indian origin (Figure 2, Tables S3, S4). The porcine strains HP113 and HP140, which exhibited the highest nucleotide sequence identity (98.8% and 98.1% respectively) to GH018-08 and GH019-08 were established to be of bovine origin [46]. Several other bovine strains of Indian, South Korean and African origin, as well as the human-artiodactyl reassortants RVA/Human-wt/HUN/BP1062/2004/G8P[14] [48] and RVA/Human-tc/ITA/PA169/1988/G6P[14] [13] clustered within the same lineage as GH018-08 and GH019-08 (Figure 2). All but two human strains clustered in a separate lineage with nucleotide sequence identities ≤93% (Table S3).

The **VP1** genes of GH018-08 and GH019-08 were distantly related (91.1% nucleotide sequence identity), clustering separately in different lineages within the R2 genotype (Figure 2, Table S3). While GH018-08 was closely related (96.1% nucleotide sequence identity) to the human-artiodactyl interspecies reassortant strain RVA/Human-wt/BEL/B1711/2002/G6P[6] [49], its next closest neighbour (91.8% nucleotide sequence identity) was the caprine strain RVA/Goat-tc/BGD/GO34/1999/G6P[1] [50]. GH018-08 was distantly related (90.2–91.6% nucleotide sequence identities) to other contemporary human strains of the same genotype, as well as the recently reported camel strain RVA/Camel-wt/SDN/MRC-DPRU447/2002/G8P[11] [51] (90.8% nucleotide sequence identity) (Figure 2, Tables S3, S4). However, GH019-08 was closely related (99.1–99.2% nucleotide sequence identity) to the contemporary human strains RVA/Human-wt/BEL/F01498/2009/G3P6, RVA/Human-wt/BEL/F01322/2009/G3P[6] and RVA/Human-wt/USA/06-242/2006/G2P[6] [52], as well as other recent human strains (97.3–98.2% nucleotide sequence identity), including many of African origin. Unlike GH018-08, GH019-08 was more closely related to RVA/Camel-wt/SDN/MRC-DPRU447/2002/G8P[11] and RVA/Goat-tc/BGD/GO34/1999/G6P[1], sharing 98.8% and 95.9% nucleotide sequence identities respectively, and clustered together in the same lineage (Figure 2, unpublished data).

The **VP2** genes of GH018-08 and GH019-08 were almost identical (99.6% nucleotide sequence identity), forming a monophyletic cluster within the C2 genotype. They were closely related to the Indian bovine strain RVA/Cow/IND/M1/UKD/2010/GXP[X], with 97.2% and 96.9%% nucleotide sequence identity respectively (Figure 2, Table S4). GH018-08 and GH019-08 revealed a closer phylogenetic relationship (89.0–89.3% nucleotide sequence identity) with Chinese ovine strains and the caprine strain RVA/Goat-tc/BGD/GO34/1999/G6P[1] compared to other artiodactyl, equine, human-artiodactyl and multiple bovine-feline/canine-human reassortant strains (86.3–87.8% nucleotide sequence identity) (Figure 2, Tables S3, S4). Human strains, however, formed a distant monophyletic cluster within the C2 genotype (Figure 2), sharing 84.4–86.6% nucleotide sequence identity with GH018-08 and GH019-08 (Table S3), very close to the proposed cut-off value of 84% [13].

The **VP3** genes of GH018-08 and GH019-08 were nearly identical (99.9% nucleotide sequence identity), and formed a monophyletic cluster within the M2 genotype. They were distantly related (90.1% nucleotide sequence identity) to their closest neighbour, the African bovine strain RVA/Bovine-tc/ZAF/‘O’;Agent/1965/G8P[1], as well as other strains of African origin - RVA/Human-tc/KEN/B12/1987/G8P[1] [53], RVA/Human-tc/KEN/D205/1989/G2P[4] [54], and RVA/Camel-wt/SDN/MRC-DPRU447/2002/G8P[11] (88.6–88.9% nucleotide sequence identity) (Figure 2, Tables S3, S4). GH018-08 and GH019-08 were more distantly related (86.7–88.0% nucleotide sequence identity) to other artiodactyl strains, and clustered separately from them (Figure 2). Human, caprine and ovine strains, including the human-artiodactyl reassortants RVA/Human-wt/HUN/Hun5/1997/G6P[14] [13] and RVA/Human-wt/HUN/BP1062/2004/G8P[14], clustered distantly in a separate lineage, with nucleotide sequence identities of 82.2-83.6% (Figure 2, Table S3).

**Figure 3:**
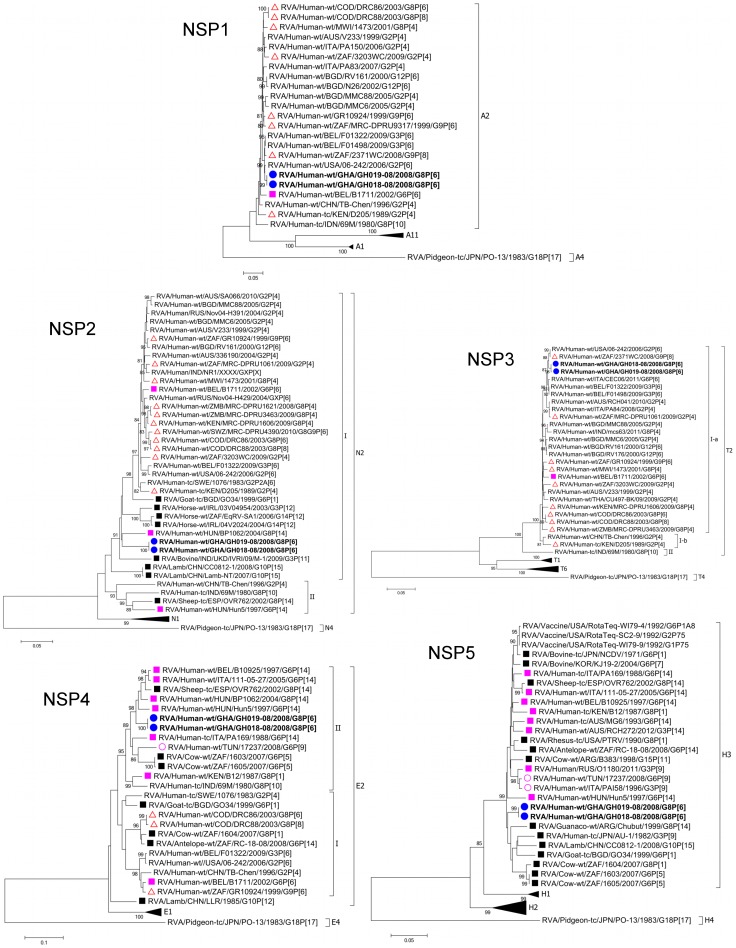
Phylograms based on the full-length open reading frame (ORF) nucleotide sequences of NSP1, NSP2, NSP3, NSP4 and NSP5 encoding genome segments of RVA strains GH018-08 and GH019-08 indicating genetic relationships with cognate RVA genes. See Legend of Figure 1.

The **NSP1** genes of GH018-08 and GH019-08 were identical, and formed a monophyletic cluster closely related (94.3-99.3% nucleotide sequence identity) to contemporary human strains within the A2 genotype (Figure 3, Tables S3, S4).

The **NSP2** genes of GH018-08 and GH019-08 were identical, forming a monophyletic cluster within the N2 genotype, and exhibited a fairly distant relationship (92.7% nucleotide sequence identity) with their closest neighbour, the human-artiodactyl reassortant strain RVA/Human-wt/HUN/BP1062/2004/G8P[14] (Figure 3, Table S3). Though GH018-08 and GH019-08 were related (87.3–92.7% nucleotide sequence identity) to human and human-artiodactyl reassortant strains, they shared just as much similarity with ovine, caprine and equine strains (87.7–90.4%, 91.8%, and 91.5–91.7% nucleotide sequence identities respectively) and clustered together (Figure 3, Tables S3, S4).

The **NSP3** genes of GH018-08 and GH019-08 were nearly identical (99.9% nucleotide sequence identity), forming a monophyletic cluster closely related (99.3–99.4% nucleotide sequence identity) to the human strains RVA/Human-wt/ZAF/2371WC/2008/G9P[8] [55] and RVA/Human-wt/USA/06-242/2006/G2P[6] within the T2 genotype (Figure 3, Tables S3, S4). GH018-08 and GH019-08 were closely related (91.0-99.4% nucleotide sequence identity) to other contemporary human strains, clustering in the same sublineage. However, RVA/Human-wt/CHN/TB-Chen/1996/G2P[4] [56] and RVA/Human-tc/KEN/D205/1989/G2P[4], sharing nucleotide sequence identities of 97.2–97.3% and 96.3–96.4% nucleotide sequence identities respectively with GH018-08 and GH019-08, clustered in a separate sublineage (Figure 3, Tables S3, S4).

The **NSP4** genes of GH018-08 and GH019-08 were identical, forming a monophyletic cluster within the E2 genotype. They revealed a fairly close relationship (93.6–94.1% nucleotide sequence identity) with the human-artiodactyl reassortants RVA/Human-wt/BEL/B10925/1997/G6P[14], RVA/Human-wt/ITA/111-05-27/2005/G6P[14] [13], RVA/Human-wt/HUN/BP1062/2004/G8P[14] and RVA/Human-wt/HUN/Hun5/1997/G6P[14], as well as the ovine strain RVA/Sheep-tc/ESP/OVR762/2002/G8P[14] [13] (93.8% nucleotide sequence identity), clustering closely within the same lineage (Figure 3, Tables S3, S4). Apart from RVA/Human-tc/IND/69M/1980/G8P[10] [12], GH018-08 and GH019-08 were more closely related (90.2–90.3% nucleotide sequence identity) to the African bovine strains RVA/Cow-wt/ZAF/1603/2007/G6P[5] and RVA/Cow-wt/ZAF/1605/2007/G6P[5] [28] compared to human strains, which shared nucleotide sequence identities ranging from borderline to just above the proposed cut-off value of 85% (84.9–86.4%), and clustered in a separate lineage (Figure 3, Tables S3, S4).

The **NSP5** genes of GH018-08 and GH019-08 were identical and formed a monophyletic cluster within the H3 genotype. They were closely related to bovine, guanaco, ovine and caprine strains (95.6–97.0%, 95.8%, 94.8–96.0%, and 95.5% nucleotide sequence identity respectively), as well as human-artiodactyl and multiple bovine-feline/canine-human reassortants (95.3–96.7% nucleotide sequence identity), clustering within the same lineage (Figure 3, Tables S3, S4). GH018-08 and GH019-08 clustered with the prototype RVA/Human-tc/JPN/AU-1/1982/G3P[9] strain of the AU-1 genogroup [57] in the same lineage (Figure 3), sharing 95.3% nucleotide sequence identity (unpublished data).

## Discussion

The determination of the complete genome sequences of the Ghanaian strains GH018-08 and GH019-08 revealed the unusual genotype constellation G8-P[6]-I2-R2-C2-M2-A2-N2-T2-E2-H3. To our knowledge, this is the first report of this genotype constellation, with a G8P[6] RVA strain possessing an H3 genotype on a DS-1-like backbone. It has previously been suggested that genotype 3 proteins tend to appear in reassortant strains carrying a predominantly genotype 2 (DS-1-like) backbone [12].

The unusually high prevalence of the common bovine genotype G8 in humans in Africa has been speculated to be the result of interspecies transmission of rotaviruses between cattle and humans [28]–[31]. The close phylogenetic relationship of the identical VP7 genes of GH018-08 and GH018-09 to both human RVA strains and a regional bovine strain (95.3–97.2% and 96% nucleotide sequence identity respectively) suggests they originated from a common ancestor and adds to the speculation on the origin of African G8 strains isolated from humans.

The P[6] genotype is epidemiologically important in Ghana, detected quite commonly in association with a wide variety of G-types [25], [32], [33], [58], [59]. The identical VP4 genes of GH018-08 and GH019-08 clustered closely with cognate genes of co-circulating Ghanaian DS-1-like strains, and showed a close phylogenetic relationship with contemporary RVA human strains, clustering in a separate lineage from strains isolated before 1994. This suggests that they may have originated from a common, contemporary progenitor strain that is established in the human population.

Despite bearing the same genotype constellation, not all genome segments of GH018-08 and GH019-08 shared close phylogenetic relationships. Of the 11 gene segments of GH018-08 and GH019-08, 10 (VP2-VP4, VP6, VP7, NSP1-NSP5) possessed identical or nearly identical (99.6–100% nucleotide sequence identity) open reading frames, forming monophyletic clusters within their respective genotypes. This suggests the presence of a recent common ancestor. However, the VP1 genes exhibited significant variation (91.1% nucleotide identity), clustering separately into different lineages within the R2 genotype. GH018-08, while forming a monophyletic cluster with the human-artiodactyl interspecies reassortant strain RVA/Human-wt/BEL/B1711/2002/G6P[6] (96.1% nucleotide sequence identity), was distantly related to other contemporary human strains, the caprine strain RVA/Goat-tc/BGD/GO34/1999/G6P[1], as well as the recently reported camel strain RVA/Camel-wt/SDN/MRC-DPRU447/2002/G8P[11] (90.2–91.6%, 91.8%, and 90.8% nucleotide sequence identity respectively), and clustered separately. On the contrary, GH019-08 was closely related to contemporary human strains, RVA/Camel-wt/SDN/MRC-DPRU447/2002/G8P[11] and RVA/Goat-tc/BGD/GO34/1999/G6P[1] (97.3–99.2%, 98.8% and 95.9% nucleotide sequence identity respectively), and clustered together in the same lineage. Considering that the VP1 gene of GH019-08 clustered within a major lineage of co-circulating DS-1-like Ghanaian strains while GH018-08 formed a unique lineage, these observations provide strong evidence of at least one recent intragenogroup reassortment event with an established locally circulating DS-1-like strain carrying a phylogenetically different VP1 gene.

Excluding VP7, VP4 and VP1, the 8 remaining genome segments forming the I2-C2-M2-A2-N2-T2-E2-H3 backbone of GH018-08 and GH019-08 could be divided into three groups based on their phylogeny – (i) segments closely related to bovine/ovine/caprine RVA strains (VP6, VP2, NSP4, and NSP5), (ii) segments distantly related to human RVA strains (VP3 and NSP2), and (iii) segments closely related to human RVA strains (NSP1and NSP3).

In the Upper East region, where the samples were collected, the human population live in close proximity with, and share common water supply with domestic animals such as goats, sheep and cattle. This highlights the potential for the occurrence of gene reassortment [36]. Bovine, ovine, and caprine rotaviruses usually bear the genotype constellation Gx-P[x]-I2-R2-C2-M2-A3/A11-N2-T6-E2-H3 [13], [50], [60]. Though GH018-08 and GH019-08 carried the conserved bovine NSP5 genotype H3, no bovine rotaviruses have as yet been found to bear P[6], A2 or T2 genotypes for their VP4, NSP1 and NSP3 genes respectively. Thus, the possibility that GH018-08 and GH019-08 resulted from a recent direct interspecies transmission of bovine rotaviruses was ruled out at genotype constellation level.

While NSP1 and NSP3, along with the VP4 genes of GH018-08 and GH019-08 were closely related to contemporary human strains, the VP6 and VP2 genes were closely related to recently isolated bovine strains, and the NSP4 and NSP5 genes were closely related to bovine/ovine/caprine strains. The NSP1- and NSP3-encoded proteins, which interact extensively with host cell factors but are not known to bind to any other viral proteins, reassort more freely than those encoding the inner capsid proteins, VP1-VP3 [12]. The VP4, NSP1 and NSP3 genes of GH018-08 and GH019-08 exhibited the closest phylogenetic relationship (99.3–99.6% nucleotide sequence identity) with the same strain, RVA/Human-wt/USA/06-242/2006/G2P[6]. Coupled with the identical (VP4, NSP1) and nearly identical (NSP3) nature of these genes in GH018-08 and GH019-08, the apparent existence of a common ancestor raises the possibility of a recent, simultaneous reassortment of all three genes into the largely bovine DS-1-like backbone. It is interesting to note that these genes, implicated in cell attachment and penetration (VP4), the evasion of the host cell innate immune response (NSP1), and enhancement of the translation of viral mRNA transcripts (NSP3) [61]–[63] were closely related to human RVA strains and shared the same common ancestor. This observation increases the significance of the observed variation in the VP1 gene, which encodes the RNA-dependent RNA polymerase, with the same common ancestor implicated in an intragenogroup reassortment event. Taken together, it is plausible to view the incorporation of human VP4, NSP1 and NSP3 genes (along with a human VP1 gene in the case of GH019-08) into a largely bovine-like genome constellation as an attempt at adaptation to a human host by an animal RVA strain. Though the origin of the NSP2 gene of GH018-08 and GH019-08 could not be clearly ascertained, it has been reported that the proteins encoded by NSP2 and NSP5 from different genotypes, and possibly different species, can functionally substitute for each other quite easily due to the conserved nature of known NSP2-NSP5 interaction sites [12]. This lends support to our hypothesis of complex attempts of an animal strain to adapt to a human host in order to survive.

Though the VP3 genes of GH018-08 and GH019-08 were distantly related to cognate rotavirus genotypes, their closest neighbour was bovine. The apparent lack of a common ancestor among the segments related to bovine, ovine or caprine strains (VP6, VP2, NSP4 and NSP5) challenges our speculation on the direction of interspecies transmission, especially since the two G8P[6] strains investigated most likely represent only a small part of a complex series of events. The availability of sequence information from local bovine species should clarify this.

Taken together, these findings suggest multiple independent interspecies transmission and reassortment events between co-circulating bovine/ovine/caprine rotaviruses and human DS-1-like RVA strains. Considering that G8 strains are only sporadically detected in Ghana, and that 10 out of the 11 genome segments of GH018-09 and GH019-08 had identical or nearly identical open reading frame nucleotide sequences, it is reasonable to suggest that while these strains might have a recent common ancestor, they are unlikely to be well adapted to humans for efficient transmission at present. These findings highlight the need for simultaneous monitoring of animal and human rotavirus strains, especially in the era of rapid spread of rotavirus vaccines in Africa.

## Supporting Information

Figure S1 **Electrophoretic migration pattern of genome segments of G8P[6]** **RVA strains GH018-08 and GH019-08, with representative Ghanaian Wa-like and DS-1-like strains.**
(PDF)

Table S1 **Sequence data for genome segments of GH018-08 and GH019-08 generated by** ***de novo*** **sequence assembly.**
(DOCX)

Table S2 **GenBank accession numbers for all rotavirus strains used for phylogenetic analyses.**
(DOCX)

Table S3 **Nucleotide sequence identities (%) of the full-length ORFs of all 11 gene segments of GH018-08 to selected human and animal reference rotavirus strains.**
(DOCX)

Table S4 **Nucleotide sequence identities (%) of the full-length ORFs of all 11 gene segments of GH018-08 to selected, relevant ‘non-reference’ human and animal rotavirus strains.**
(DOCX)
