# Supplementary material for: Identification of Novel Ghanaian G8P[6] Human-Bovine Reassortant Rotavirus Strain by Next Generation Sequencing
Source: PLoS One. 2014 Jun 27;9(6):e100699. doi: 10.1371/journal.pone.0100699 (PMC4074113; doi:10.1371/journal.pone.0100699)
Supplement: Figure S1 — Electrophoretic migration pattern of genome segments of G8P[6] RVA strains GH018-08 and GH019-08, with representative Ghanaian Wa-like and DS-1-like strains. (PDF) [file pone.0100699.s001.pdf]

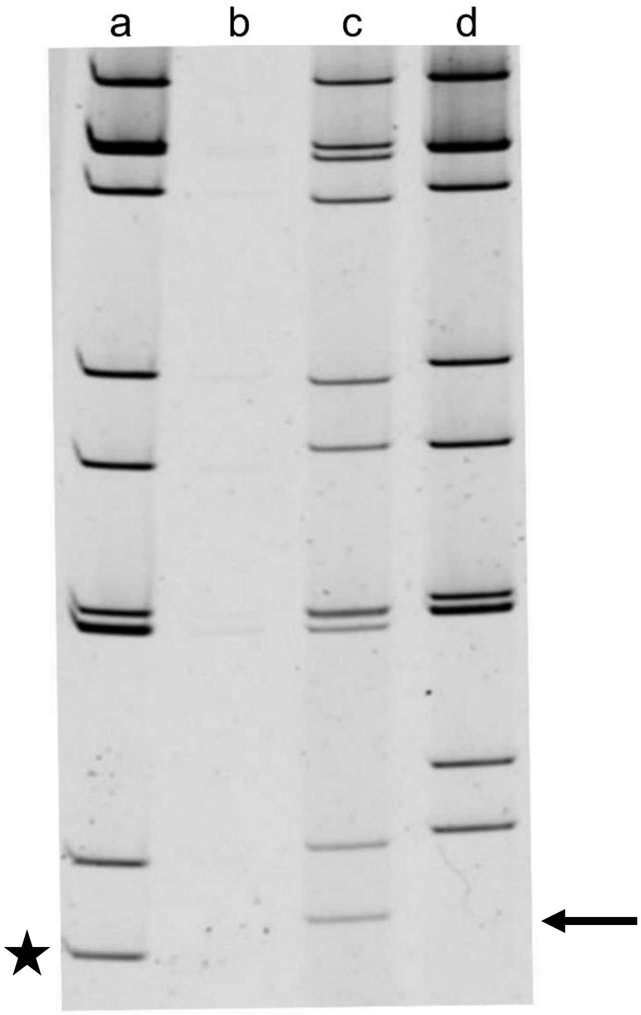

Figure S1. Electrophoretic migration pattern of genome segments of G8P[6] RVA strains GH018-08 and GH019-08, with representative Ghanaian Wa-like and DS-1-like strains.

Lanes: a) GH018-08; b) GH019-08; c) GH022-08; d) GH024-08 (GH022-08: G1P[8] Wa-like strain; GH024-08: G2P[4] DS-1-like strain.)

GH018-08 and GH019-08 revealed identical long RNA migration patterns, with a notably longer migration distance for gene segment 11 (NSP5) than observed in Wa-like RVA strains (indicated by star and arrow respectively.
