# Supplementary material for: Identification of Novel Ghanaian G8P[6] Human-Bovine Reassortant Rotavirus Strain by Next Generation Sequencing
Source: PLoS One. 2014 Jun 27;9(6):e100699. doi: 10.1371/journal.pone.0100699 (PMC4074113; doi:10.1371/journal.pone.0100699)
Supplement: Table S1 — Sequence data for genome segments of GH018-08 and GH019-08 generated by de novo sequence assembly. (DOCX) [file pone.0100699.s002.docx]

Table S1. Sequence data for genome segments of GH018-08 and GH019-08 generated by *de novo* sequence assembly

| Strain | Total Reads^a^ | Sequence Reads^a^ | Genome segments | | | | | | | | | | | |
| --- | --- | --- | --- | --- | --- | --- | --- | --- | --- | --- | --- | --- | --- | --- |
|  |  |  | VP1 | VP2^b^ | VP3 | VP4 | VP6 | VP7 | NSP1 | NSP2 | NSP3 | NSP4 | NSP5 | NSP6 |
| *Nucleotide (bp)* |  |  | *3301* | *2690* | *2591* | *2359* | *1355* | *1062* | *1565* | *1050* | *1066* | *750* | *661* | *661* |
| **RVA/Human-wt/GHA/GH018-08/2008/G8P[6]** | 1, 472, 401 | Total reads mapped to each segment | 236,542 | 236,640 | 212,516 | 224,152 | 54,947 | 73,682 | 81,988 | 79,648 | 91,658 | 77,465 | 14,711 |  |
|  |  | Maximum depth of reads | 11,072 | 15,936 | 12,217 | 14,654 | 10,722 | 12,006 | 17,054 | 9,003 | 15,274 | 19,890 | 4,400 |  |
|  |  |  |  |  |  |  |  |  |  |  |  |  |  |  |
| *Nucleotide (bp)* |  |  | *3302* | *2688* | *2588* | *2359* | *1356* | *1058* | *1566* | *1058* | *1066* | *748* | *661* | *661* |
| **RVA/Human-wt/GHA/GH019-08/2008/G8P[6]** | 1, 160, 020 | Total reads mapped to each segment | 71,440 | 68,476 | 72,050 | 54,697 | 32,260 | 21,518 | 41,261 | 65,581 | 67,613 | 15,940 | 9,091 |  |
|  |  | Maximum depth of reads | 2,817 | 6,290 | 4,125 | 5,113 | 7,293 | 5,264 | 7,770 | 8,870 | 9,277 | 6,400 | 3,641 |  |
|  |  |  |  |  |  |  |  |  |  |  |  |  |  |  |
| *Deduced amino acids (aa)* |  |  |  |  |  |  |  |  |  |  |  |  |  |  |
| RVA/Human-wt/GHA/GH018-08/2008/G8P[6] |  |  | 1088 | *881* | 835 | 775 | 397 | 326 | 493 | 317 | 313 | 175 | 198 | 98 |
| RVA/Human-wt/GHA/GH019-08/2008/G8P[6] |  |  | 1088 | *881* | 835 | 775 | 397 | 326 | 493 | 317 | 313 | 175 | 198 | 98 |
|  |  |  |  |  |  |  |  |  |  |  |  |  |  |  |

^a^Sequence reads remaining after adapter trimming

^b^Unusual lengths for the VP2 genes (nucleotide and amino acid) for GH018-08 and GH019-08 are shown in red, italicized font
