# Supplementary material for: Identification of Novel Ghanaian G8P[6] Human-Bovine Reassortant Rotavirus Strain by Next Generation Sequencing
Source: PLoS One. 2014 Jun 27;9(6):e100699. doi: 10.1371/journal.pone.0100699 (PMC4074113; doi:10.1371/journal.pone.0100699)
Supplement: Table S2 — GenBank accession numbers for all rotavirus strains used for phylogenetic analyses. (DOCX) [file pone.0100699.s003.docx]

Table S2. GenBank accession numbers for all rotavirus strains used for phylogenetic analyses

| **A. VP7** | | | | |
| --- | --- | --- | --- | --- |
| Strain | Accession Number |  | Strain | Accession Number |
| RVA/Human-wt/JPN/Hochi/1980/G4P[8] | AB039035.1 |  | RVA/Human-wt/KEN/KY6914/2002/G8P[4] | FJ386445.1 |
| RVA/Human-tc/GBR/ST3/1975/G4P2A[6] | EF672616.1 |  | RVA/Human-wt/KEN/KY6950/2002/G8P[6] | FJ386446.1 |
| RVA/Human/ZAF/GR1106/86/XXXX/G4P[X] | AF170837.1 |  | RVA/Bovine-tc/NGR/NGRBg8/1998/G8P[1] | AF361439.1 |
| RVA/Human-wt/BGD/Dhaka16/2003/G1P[8] | DQ492674.1 |  | RVA/Human-wt/ZAF/2371WC/2008/G9P[8] | JN014000.1 |
| RVA/Human-wt/THA/CMH042/2004/G1P[8] | EF199713.1 |  | RVA/Human-wt/ZAF/GR570/85/1987/G8P[X] | AF143688.1 |
| RVA/Human-wt/CHN/TB-Chen/1996/G2P[4] | AY787646.1 |  | RVA/Bovine-wt/IND/68/2007/G8P[14] | GU984760.1 |
| RVA/Human-wt/USA/06-242/2006/G2P[6] | JF460817.1 |  | RVA/Bovine-wt/IND/86/2007/G8P[14] | GU984762.1 |
| RVA/Vaccine/USA/1290xUK/2003/G8P[5] | GQ225781.1 |  | RVA/Human-wt/HUN/BP1062/2004/G8P[14] | FN665696.1 |
| RVA/Vaccine/USA/MVS-BRV1290xUK/2005/G8P[5] | KC215513.1 |  | RVA/Bovine-wt/EGY/EGY2022/2011/G8P[14] | KF305321.1 |
| RVA/Vaccine/USA/BRV1290G8xUK/2003/G8P[5] | GQ496277.1 |  | RVA/Rhesus-tc/USA/PTRV/1990/G8P[1] | FJ422138.1 |
| RVA/Vaccine/USA/LMVS-BRV1290xUK/2003/G8P[5] | GQ496267.1 |  | RVA/Human-tc/KEN/B12/1987/G8P[1] | HM627547.1 |
| RVA/Vaccine/USA/ROT-BRV1290xUK/2003/G8P[5] | GQ496281.1 |  | RVA/Sheep-tc/ESP/OVR762/2002/G8P[14] | EF554153.1 |
| RVA/Human/1290/Kenya/1991/G8P[X] | EU488721.1 |  | RVA/Human-tc/IND/69M/1980/G8P[10] | EF672560.1 |
| RVA/Human-wt/MWI/MW4097/2000/G8P[8] | FJ386442.1 |  | RVA/Cow-wt/ZAF/1604/2007/G8P[1] | JN831225.1 |
| RVA/Human-wt/MWI/MW4103/2000/G8P[8] | FJ386443.1 |  | RVA/Human-wt/DEU/GER1H-09/2009/G8P[4] | GQ414545.1 |
| RVA/Human-tc/MWI/MW333/1997/G8P[4] | AJ278257.1 |  | RVA/Human-wt/HUN/Hun5/1997/G6P[14] | EF554109.1 |
| RVA/Human-wt/MW1479/2001/G8P[4] | FJ386441.1 |  | RVA/Goat-tc/BGD/GO34/1999/G6P[1] | GU937882.1 |
| RVA/Human-wt/MWI/1473/2001/G8P[4] | HQ657143.1 |  | RVA/Human-wt/BEL/B1711/2002/G6P[6] | EF554087.1 |
| RVA/Human-wt/MW1470/2001/G8P[4] | FJ386440.1 |  | RVA/Human/GHA/402/200X/G10P[6] | AY843332.2 |
| RVA/Human-tc/MWI/MW23/1997/G8P[6] | AJ278254.1 |  | RVA/Human/GHA/86/200X/G10P[6] | AY843333.2 |
| RVA/Human-wt/COD/DRC86/2003/G8P[6] | DQ005120.1 |  | RVA/Human/CIV/1784CI/99/1999/G10P[8] | AY816181.1 |
| RVA/Human-wt/SWZ/MRC-DPRU4390/2010/G8G9P[6] | KF636351.1 |  | RVA/Human-wt/AUS/V585/2011/G10P[14] | JX567749.1 |
| RVA/Human-wt/COD/DRC88/2003/G8P[8] | DQ005109.1 |  | RVA/Human-wt/BEL/F01322/2009/G3P[6] | JF460828.1 |
| RVA/Simian/KEN/KY1646/1999/G8P[6] | FJ386444.1 |  | RVA/Human-wt/GHA/GH1319/XXXX/G9P[6] | AY211065.1 |
| RVA/Human/MWI/MAL81XXXX//G8P[4] | JN591405.1 |  | RVA/Human-wt/BEL/B3458/2003/G9P[8] | EF990708.1 |
| RVA/Human-wt/ZMB/MRC-DPRU1621/2008/G8P[4] | KF636362.1 |  | RVA/Human-wt/ZAF/GR10924/1999/G9P[6] | FJ183360.1 |
| RVA/Human-wt/ZMB/MRC-DPRU3463/2009/G8P[4] | KF636316.1 |  | RVA/Human-wt/BEL/B4633/2003/G12P[8] | DQ146643.1 |
| RVA/Human-wt/KEN/MRC-DPRU1606/2009/G8P[4] | KF636250.1 |  | RVA/Human-wt/ZAF/3176WC/2009/G12P[6] | HQ657165.1 |
| RVA/Human/NGR/HMG89/XXXX/G8P[X] | X98918.1 |  | RVA/Pigeon-tc/JPN/PO-13/1983/G18P[17] | D82979.1 |
| **B. VP4** | | | | |
| Strain | Accession Number |  | Strain | Accession Number |
| RVA/Human-wt/HUN/BP[10]62/2004/G8P[14] | FN665691.1 |  | RVA/Human-wt/ZAF/2371WC/2008/G9P[8] | JN013997.1 |
| RVA/Sheep-tc/ESP/OVR762/2002/G8P[14] | EF554151.1 |  | RVA/Human-wt/BGD/RV161/2000/G12P[6] | DQ490548.1 |
| RVA/Human-wt/HUN/Hun5/1997/G6P[14] | EF554107.1 |  | RVA/Human-wt/BGD/RV176/2000/G12P[6] | DQ490554.1 |
| RVA/Human-wt/BGD/Dhaka16/2003/G1P[8] | DQ492672.1 |  | RVA/Human-wt/ZAF/MRC-DPRU9317/1999/G9P[6] | JN605440.1 |
| RVA/Human-wt/BEL/B3458/2003/G9P[8] | EF990707.1 |  | RVA/Human/KOR/CAU214/G12P[6] | EF059921.1 |
| RVA/Human-wt/BEL/B4633/2003/G12P[8] | DQ146641.1 |  | RVA/Human/ZAF/GR10924/99/1999/G9P[6] | FJ183356.1 |
| RVA/Human-wt/COD/DRC88/2003/G8P[8] | DQ005111.1 |  | RVA/Human/BGD/SK277/2005/G12P[6] | EU839948.1 |
| RVA/Human/CHN/TB-Chen/1996/G2P[4] | AY787644.1 |  | RVA/Human-wt/ZAF/3176WC/2009/G12P[6] | HQ657163.1 |
| RVA/Human/BGD/MMC88/2005/G2P[4] | HQ641373.1 |  | RVA/Human/CHN/XJ99-468/1999/G9P[6] | DQ321496.1 |
| RVA/Human-wt/COD/DRC86/2003/G8P[6] | DQ005122.1 |  | RVA/Human/AUS/RV3/1993/G3P[6] | U16299.1 |
| RVA/Human-wt/SWZ/MRC-DPRU4390/2010/G8G9P[6] | KF636348.1 |  | RVA/Human-tc/AUS/RV3/1993/G3P[6] | FJ998273.1 |
| RVA/Human/MWI/MW23/1997/G8P[6] | AJ278253.1 |  | RVA/Human-tc/GBR/ST3/1975/G4P[6] | L33895.1 |
| RVA/Human/BEL/F01322/2009/G3P[6] | JF460826.1 |  | RVA/Human-tc/SWE/1076/1983/G2P2A[6] | M88480.1 |
| RVA/Human/BEL/F01498/2009/G3P[6] | JF460837.1 |  | RVA/Human-tc/IDN/69M/1980/G8P[10] | M60600.1 |
| RVA/Human-wt/BEL/B1711/2002/G6P[6] | EF554085.1 |  | RVA/Goat-tc/BGD/GO34/1999/G6P[1] | GU937880.1 |
| RVA/Human/USA/06-242/2006/G2P[6] | JF460815.1 |  | RVA/Pidgeon-tc/JPN/PO-13/1983/G18P[17] | AB009632.2 |
| RVA/Human-wt/ZAF/2371WC/2008/G9P[8] | JN013996.1 |  |  |  |

Table S2. Continued

| **C. VP6** | | | | |
| --- | --- | --- | --- | --- |
| Strain | Accession Number |  | Strain | Accession Number |
| RVA/Human-wt/BEL/B4633/2003/G12P[8] | DQ146642.1 |  | RVA/Antelope-wt/ZAF/RC-18-08/2008/G6P[14] | FJ495131.1 |
| RVA/Human-tc/GBR/ST3/1975/G4P2A[6] | EF583048.1 |  | RVA/Human-wt/ZAF/2371WC/2008/G9P[8] | JN014002.1 |
| RVA/Human-wt/BGD/Dhaka16/2003/G1P[8] | DQ492673.1 |  | RVA/Sheep-tc/ESP/OVR762/2002/G8P[14] | EF554152.1 |
| RVA/Human-wt/BEL/B3458/2003/G9P[8] | DQ870504.1 |  | RVA/Bovine/KOR/KJ9-1/2006/G6P[7] | HM988974.1 |
| RVA/Cow/IND/970/2009/G3P[X] | HQ171911.1 |  | RVA/Vaccine/USA/RotaTeq-WI79-4/1992/G6P1A[8] | GU565045.1 |
| RVA/Bovine/IND/UKD/P[14]/2009/G3P[1] | JF742650.1 |  | RVA/Human-tc/ITA/PA169/1988/G6P[14] | EF554130.1 |
| RVA/Porcine-wt/IND/HP[14]0/XXXX/G6P[13] | DQ003295.1 |  | RVA/Human-wt/RUS/Nov10-N539/2010/G2P[4] | HQ611024.2 |
| RVA/Porcine-wt/IND/HP113/XXXX/G6P[13] | DQ003294.1 |  | RVA/Human-tc/IND/69M/1980/G8P[10] | EF583016.1 |
| RVA/Bovine-wt/IND/86/2007/G8P[14] | GU984759.1 |  | RVA/Human-wt/BEL/F01322/2009/G3P[6] | JF460827.1 |
| RVA/Bovine-wt/IND/68/2007/G8P[14] | GU984757.1 |  | RVA/Human-wt/USA/06-242/2006/G2P[6] | JF460816.1 |
| RVA/Bovine-wt/IND/79/2007/G8P[14] | GU984758.1 |  | RVA/Human-wt/BEL/B1711/2002/G6P[6] | EF554086.1 |
| RVA/Bovine-wt/IND/HR/BRV133/2008/G3P[1] | JF720875.1 |  | RVA/Human-wt/BGD/RV161/2000/G12P[6] | DQ490549.1 |
| RVA/Bovine-wt/IND/UP/Bov1/2009/GXP[X] | JF742649.1 |  | RVA/Human-wt/COD/DRC86/2003/G8P[6] | DQ005121.1 |
| RVA/Bovine-wt/IND/HR/CC156/2010/G3P[X] | JF720879.1 |  | RVA/Human-wt/COD/DRC88/2003/G8P[8] | DQ005110.1 |
| RVA/Cow/IND/Bov2/UP/2008/GXP[X] | HQ440218.1 |  | RVA/Human-wt/ZAF/GR10924/1999/G9P[6] | FJ183358.1 |
| RVA/Cow/IND/B-72/2008/G10P[X] | HQ171913.1 |  | RVA/Goat-tc/BGD/GO34/1999/G6P[1] | GU937881.1 |
| RVA/Bovine-wt/IND/UKD/09/M-1/2009/G3P[11] | HM235508.1 |  | RVA/Human-wt/HUN/Hun5/1997/G6P[14] | EF554108.1 |
| RVA/Human-wt/HUN/BP1062/2004/G8P[14] | FN665693.1 |  | RVA/Human-wt/CHN/TB-Chen/1996/G2P4 | AY787645.1 |
| RVA/Cow-wt/ZAF/1604/2007/G8P[1] | JN831224.1 |  | RVA/Pidgeon-tc/JPN/PO-13/1983/G18P[17] | D16329.2 |
| RVA/Cow/ZAF/MRC-DPRU1604/2007/G6P[1] | KF636260.1 |  |  |  |
| **D. VP1** | | | | |
| Strain | Accession Number |  | Strain | Accession Number |
| RVA/Human-wt/BGD/Dhaka16/2003/G1P[8] | DQ492669.1 |  | RVA/Human-wt/SWZ/MRC-DPRU4390/2010/G8G9P[6] | KF636345.1 |
| RVA/Human-wt/BEL/B3458/2003/G9P[8] | DQ870501.1 |  | RVA/Human-wt/COD/DRC88/2003/G8P[8] | DQ005114.1 |
| RVA/Human-wt/BEL/B4633/2003/G12P[8] | DQ146638.1 |  | RVA/Human-wt/ZMB/MRC-DPRU1621/2008/G8P[4] | KF636357.1 |
| RVA/Human-wt/ZAF/3176WC/2009/G12P[6] | HQ657160.1 |  | RVA/Human-wt/ZMB/MRC-DPRU3463/2009/G8P[4] | KF636311.1 |
| RVA/Human-tc/GBR/ST3/1975/G4P2A[6] | EF583045.1 |  | RVA/Human-wt/KEN/MRC-DPRU1606/2009/G8P[4] | KF636245.1 |
| RVA/Human/ZAF/GR/10924/99/1999/G9P[6] | FJ183353.1 |  | RVA/Human-wt/BGD/RV161/2000/G12P[6] | DQ490545.1 |
| RVA/Human-wt/ZAF/MRC-DPRU9317/1999/G9P[6] | JN605437.1 |  | RVA/Human-wt/BGD/RV176/2000/G12P[6] | DQ490551.1 |
| RVA/Human-wt/THA/CU473-BK/09/2009/G2P[4] | JN706465.1 |  | RVA/Human-wt/BGD/N26/2002/G12P[6] | DQ146682.1 |
| RVA/Human-wt/USA/VU05-06-66/2005/G2P[4] | KC442985.1 |  | Human-wt/RVA/BGD/MMC6/2005/G2P[4] | HQ641355.1 |
| RVA/Human-wt/ITA/PA150/2006/G2P[4] | KC178766.1 |  | RVA/Human/BGD/MMC88/2005/G2P[4] | HQ641364.1 |
| RVA/Human-wt/AUS/V233/1999/G2P[4] | KC834713.1 |  | RVA/Goat-tc/BGD/GO34/1999/G6P[1] | GU937877.1 |
| RVA/Human-wt/ZAF/3203WC/2009/G2P[4] | HQ657171.1 |  | RVA/Human-wt/ITA/PA83/2007/G2P[4] | KC178767.1 |
| RVA/Human-wt/BEL/F01322/2009/G3P[6] | JF460823.1 |  | RVA/Human-wt/AUS/MON008/2010/G2P[4] | JX965135.1 |
| RVA/Human-wt//BEL/F01498/2009/G3P[6] | JF460834.1 |  | RVA/Human-wt/BEL/B1711/2002/G6P[6] | EF554082.1 |
| RVA/Camel-wt/SDN/MRC-DPRU447/2002/G8P[11] | KC257091.1 |  | RVA/Human-wt/CHN/TB-Chen/1996/G2P[4] | AY787653.1 |
| RVA/Human-wt/USA/06-242/2006/G2P[6] | JF460812.1 |  | RVA/Human-wt/HUN/BP1062/2004/G8P[14] | FN665688.1 |
| RVA/Human-wt/ITA/PA84/2008/G2P[4] | KC178770.1 |  | RVA/Human-tc/IND/69M/1980/G8P[10] | EF576937.1 |
| RVA/Human-wt/USA/2007769964/2007/G2P[4] | KC442903.1 |  | RVA/Sheep-tc/ESP/OVR762/2002/G8P[14] | EF554148.1 |
| RVA/Human-wt/AUS/RCH041/2010/G2P[4] | JX965134.1 |  | RVA/Human-wt/HUN/Hun5/1997/G6P[14] | EF554104.1 |
| RVA/Human-wt/ZAF/MRC-DPRU1061/2009/G2P[4] | KF636322.1 |  | RVA/Pidgeon-tc/JPN/PO-13/1983/G18P[17] | AB009629.2 |
| RVA/Human-wt/COD/DRC86/2003/G8P[6] | DQ005125.1 |  |  |  |

Table S2. Continued

| **E. VP2** | | | | |
| --- | --- | --- | --- | --- |
| Strain | Accession Number |  | Strain | Accession Number |
| RVA/Human-wt/BGD/Dhaka16/2003/G1P[8] | DQ492670.1 |  | RVA/Guanaco-wt/ARG/Chubut/1999/G8P[14] | FJ347101.1 |
| RVA/Human-wt/BEL/B3458/2003/G9P[8] | DQ870502.1 |  | RVA/Guanaco-wt/ARG/Rio/Negro/1998/G8P[1] | FJ347123.1 |
| RVA/Human-wt/BEL/B4633/2003/G12P[8] | DQ146639.1 |  | RVA/Rhesus-tc/USA/PTRV/1990/G8P[1] | FJ422132.1 |
| RVA/Human-tc/GBR/ST3/1975/G4P2A[6] | EF583046.1 |  | RVA/Bovine-tc/ZAF/O/Agent/1965/G8P[1] | JF693038.1 |
| RVA/Human-tc/USA/Se584/1998/G6P[9] | EF583042.1 |  | RVA/Human-tc/KEN/B12/1987/G8P[1] | HM627543.1 |
| RVA/Human-wt/ITA/PAI58/1996/G3P[9] | GU296423.1 |  | RVA/Horse-wt/IRL/03V04954/2003/G3P[12] | JN903526.1 |
| RVA/Human-wt/TUN/17237/2008/G6P[9] | JX271002.1 |  | RVA/Human-wt/HUN/BP1062/2004/G8P[14] | FN665689.1 |
| RVA/Human-tc/AUS/MG6/1993/G6P[14] | EF554094.1 |  | RVA/Bovine/CHN/DQ-75/2008/G10P[11] | GU384191.1 |
| RVA/Cow-wt/ZAF/1603/2007/G6P[5] | JN831210.1 |  | RVA/Lamb/CHN/LLR/1985/G10P[12] | JQ013503.1 |
| RVA/Cow-wt/ZAF/1605/2007/G6P[5] | JN831232.1 |  | RVA/Lamb/CHN/CC0812-1/2008/G10P[15] | HQ834198.1 |
| RVA/Antelope-wt/ZAF/RC-18-08/2008/G6P[14] | FJ495127.1 |  | RVA/Lamb/CHN/Lamb-NT/2007/G10P[15] | FJ031025.1 |
| RVA/Human-wt/HUN/Hun5/1997/G6P[14] | EF554105.1 |  | RVA/Caprine/CHN/XL/2010/G10P[15] | JQ004971.1 |
| RVA/Horse-tc/JPN/OH-4/1982/G6P[5] | KC815659.1 |  | RVA/Goat-tc/BGD/GO34/1999/G6P[1] | GU937878.1 |
| RVA/Cow-tc/USA/WC3/1981/G6P[5] | EF560616.1 |  | RVA/Cow/IND/M1/UKD/2010/GXP[X] | HQ440221.1 |
| RVA/Cow-tc/USA/NCDV/1967/G6P[1] | DQ870494.1 |  | RVA/Human-wt/USA/06-242/2006/G2P[6] | JF460813.1 |
| RVA/Bovine-tc/USA/NCDV/1971/G6P[1] | JF693027.1 |  | RVA/Human-wt/BEL/F01322/2009/G3P[6] | JF460824.1 |
| RVA/Human-wt/IND/N37/2003/G10P[11] | KC175084.1 |  | RVA/Human-wt/BEL/B1711/2002/G6P[6] | EF554083.1 |
| RVA/Cat-wt/ITA/BA222/2005/G3P[9] | GU827407.1 |  | RVA/Human-wt/COD/DRC86/2003/G8P[6] | DQ005124.1 |
| RVA/Human-tc/IND/69M/1980/G8P[10] | EF583014.1 |  | RVA/Human-wt/COD/DRC88/2003/G8P[8] | DQ005113.1 |
| RVA/Human-wt/HUN/BP1879/2003/G6P[14] | FN665678.1 |  | RVA/Human/ZAF/GR_10924/99/1999/G9P[6] | FJ183354.1 |
| RVA/Rabbit-tc/ITA/30-96/1996/G3P[14] | DQ205222.1 |  | RVA/Human-wt/BGD/RV161/2000/G12P[6] | DQ490546.1 |
| RVA/Human-wt/BEL/B4106/2000/G3P[14] | AY740740.1 |  | RVA/Human-wt/CHN/TB-Chen/1996/G2P[4] | AY787652.1 |
| RVA/Human-wt/ITA/111-05-27/2005/G6P[14] | EF554138.1 |  | RVA/Pidgeon-tc/JPN/PO-13/1983/G18P[17] | AB009630.2 |
| RVA/Sheep-tc/ESP/OVR762/2002/G8P[14] | EF554149.1 |  |  |  |
| **F. VP3** | | | | |
| Strain | Accession Number |  | Strain | Accession Number |
| RVA/Human-wt/BGD/Dhaka16/2003/G1P[8] | DQ492671.1 |  | RVA/Vaccine/USA/RotaTeq-WI79-4/1992/G6P1A[8] | GU565043.1 |
| RVA/Human-tc/GBR/ST3/1975/G4P2A[6] | AY277919.1 |  | RVA/Guanaco-wt/ARG/Rio/Negro/1998/G8P[1] | FJ347124.1 |
| RVA/Human-wt/BEL/B3458/2003/G9P[8] | DQ870503.1 |  | RVA/Antelope-wt/ZAF/RC-18-08/G6P[14] | FJ495128.1 |
| RVA/Human-wt/BEL/B4633/2003/G12P[8] | DQ146640.1 |  | RVA/Human-wt/BEL/B1711/2002/G6P[6] | EF554084.1 |
| RVA/Human-wt/JPN/Hochi/1980/G4P[8] | AY277915.1 |  | RVA/Human-tc/KEN/D205/1989/G2P[4] | JF304917.1 |
| RVA/Vaccine/USA/MVS-BRV1290xUK/2005/G8P[5] | KC215510.1 |  | RVA/Camel-wt/SDN/MRC-DPRU447/2002/G8P[11] | KC257093.1 |
| RVA/Vaccine/USA/MVS-BRV4/1998/G4P[5] | KC215499.1 |  | RVA/Bovine-tc/ZAF/O_Agent/1965/G8P[1] | JF693039.1 |
| RVA/Bovine/GBR/UKtc/XXXX/G6P[5] | AY300923.1 |  | RVA/Human-tc/KEN/B12/1987/G8P[1] | HM627544.1 |
| RVA/Bovine-tc/GBR/CP-1/1973/G3P[5] | FJ560906.1 |  | RVA/Human-wt/BEL/F01322/2009/G3P[6] | JF460825.1 |
| RVA/Cow-tc/USA/NCDV/1967/G6P[1] | DQ870495.1 |  | RVA/Human-wt/USA/06-242/2006/G2P[6] | JF460814.1 |
| RVA/Bovine/FRA/RF/1982/G6P[1] | AY116592.1 |  | RVA/Human-wt/COD/DRC86/2003/G8P[6] | DQ005123.1 |
| RVA/Bovine/KOR/KJ25-1/200X/G8P[7] | HM988962.1 |  | RVA/Human-wt/COD/DRC88/2003/G8P[8] | DQ005112.1 |
| RVA/Bovine/KOR/KJ56-1/2004/G8P[7] | KF500176.1 |  | RVA/Human-wt/ZAF/GR/10924/99/1999/G9P[6] | FJ183355.1 |
| RVA/Porcine/KOR/174-1/2006/G8P[7] | KF500209.1 |  | RVA/Human-wt/BGD/RV161/2000/G12P[6] | DQ490547.1 |
| RVA/Porcine/KOR/42-1/2006/G8P[7] | KF500187.1 |  | RVA/Human-wt/CHN/TB-Chen/1996/G2P[4] | AY787654.1 |
| RVA/Rhesus-tc/USA/PTRV/1990/G8P[1] | FJ422133.1 |  | RVA/Sheep-tc/ESP/OVR762/2002/G8P[14] | EF554150.1 |
| RVA/Human-tc/IDN/69M/1980/G8P[10] | AY277916.1 |  | RVA/Human-wt/HUN/Hun5/1997/G6P[14] | EF554106.1 |
| RVA/Vaccine/USA/RotaTeq-WI78-8/1992/G3P7[5] | GU565076.1 |  | RVA/Human-wt/HUN/BP1062/2004/G8P[14] | FN665690.1 |
| RVA/Cow-tc/USA/WC3/1981/G6P[5] | EF560617.1 |  | RVA/Goat-tc/BGD/GO34/1999/G6P[1] | GU937879.1 |
| RVA/Vaccine/USA/RotaTeq-BrB-9/1996/G4P7[5] | GU565087.1 |  | RVA/Pidgeon-tc/JPN/PO-13/1983/G18P[17] | AB009631.2 |

Table S2. Continued

| **G. NSP1** | | | | |
| --- | --- | --- | --- | --- |
| Strain | Accession Number |  | Strain | Accession Number |
| RVA/Human-wt/BEL/F01322/2009/G3P[6] | JF460829.1 |  | RVA/Human-wt/HUN/BP1062/2004/G8P[14] | FN665692.1 |
| RVA/Human-wt/BEL/F01498/2009/G3P[6] | JF460840.1 |  | RVA/Human-tc/GBR/ST3/1975/G4P2A[6] | EF672613.1 |
| RVA/Human-wt/ZAF/2371WC/2008/G9P[8] | JN013974.1 |  | RVA/Human-wt/BGD/Dhaka16/2003/G1P[8] | DQ492675.1 |
| RVA/Human-wt/USA/06-242/2006/G2P[6] | JF460818.1 |  | RVA/Human-wt/BEL/B3458/2003/G9P[8] | EF990709.1 |
| RVA/Human-wt/COD/DRC86/2003/G8P[6] | DQ005119.1 |  | RVA/Human-wt/BEL/B4633/2003/G12P[8] | DQ146644.1 |
| RVA/Human-wt/COD/DRC88/2003/G8P[8] | DQ005108.1 |  | RVA/Human-tc/IDN/69M/1980/G8P[10] | D38151.1 |
| RVA/Human-wt/BGD/RV161/2000/G12P[6] | DQ490540.1 |  | RVA/Human-tc/KEN/D205/1989/G2P[4] | JF304921.1 |
| RVA/Human-wt/BGD/N26/2002/G12P[6] | DQ146688.1 |  | RVA/Human-wt/CHN/TB-Chen/1996/G2P[4] | AY787647.1 |
| RVA/Human-wt/ITA/PA83/2007/G2P[4] | KC178723.1 |  | RVA/Human-wt/BEL/B1711/2002/G6P[6] | EF554088.1 |
| RVA/Human-wt/ITA/PA150/2006/G2P[4] | KC178722.1 |  | RVA/Human-wt/MWI/1473/2001/G8P[4] | HQ657133.1 |
| RVA/Human-wt/ZAF/3203WC/2009/G2P[4] | HQ657166.1 |  | RVA/Human-wt/BGD/MMC88/2005/G2P[4] | HQ641368.1 |
| RVA/Human-wt/AUS/V233/1999/G2P[4] | KC834698.1 |  | RVA/Human-wt/BGD/MMC6/2005/G2P[4] | HQ641359.1 |
| RVA/Sheep-tc/ESP/OVR762/2002/G8P[14] | EF554154.1 |  | RVA/Human-wt/GR10924/1999/G9P[6] | FJ183357.1 |
| RVA/Goat-tc/BGD/GO34/1999/G6P[1] | GU937883.1 |  | RVA/Human-wt/ZAF/MRC-DPRU9317/1999/G9P[6] | JN605443.1 |
| RVA/Human-wt/HUN/Hun5/1997/G6P[14] | EF554110.1 |  | RVA/Pidgeon-tc/JPN/PO-13/1983/G18P[17] | AB009633.2 |
| **H. NSP2** | | | | |
| Strain | Accession Number |  | Strain | Accession Number |
| RVA/Human-wt/BGD/Dhaka16/2003/G1P[8] | DQ492676.1 |  | RVA/Human-wt/ZMB/MRC-DPRU1621/2008/G8P[4] | KF636353.1 |
| RVA/Human-wt/BEL/B4633/2003/G12P[8] | DQ146645.1 |  | RVA/Human-wt/ZMB/MRC-DPRU3463/2009/G8P[4] | KF636307.1 |
| RVA/Human-tc/GBR/ST3/1975/G4P2A[6] | EF672615.1 |  | RVA/Human-wt/KEN/MRC-DPRU1606/2009/G8P[4] | KF636241.1 |
| RVA/Human-wt/BEL/B3458/2003/G9P[8] | EF990710.1 |  | RVA/Human-wt/COD/DRC86/2003/G8P[6] | DQ005118.1 |
| RVA/Lamb/CHN/CC0812-1/2008/G10P[15] | HQ834203.1 |  | RVA/Human-wt/COD/DRC88/2003/G8P[8] | DQ005107.1 |
| RVA/Lamb/CHN/Lamb-NT/2007/G10P[15] | FJ031020.1 |  | RVA/Human-wt/SWZ/MRC-DPRU4390/2010/G8G9P[6] | KF636341.1 |
| RVA/Horse-wt/ZAF/EqRV-SA1/2006/G14P[12] | JQ345497.1 |  | RVA/Human-wt/RUS/Nov04-H429/2004/P[6] | KC155671.1 |
| RVA/Horse-wt/IRL/04V2024/2004/G14P[12] | JN903513.2 |  | RVA/Human-wt/BEL/B1711/2002/G6P[6] | EF554089.1 |
| RVA/Horse-wt/IRL/03V04954/2003/G3P[12] | JN903514.2 |  | RVA/Human-wt/BEL/F01322/2009/G3P[6] | JF460830.1 |
| RVA/Human-wt/HUN/BP1062/2004/G8P[14] | FN665694.1 |  | RVA/Human-wt/USA/06-242/2006/G2P[6] | JF460819.1 |
| RVA/Human-wt/ZAF/MRC-DPRU1061/2009/G2P[4] | KF636318.1 |  | RVA/Human-wt/ZAF/3203WC/2009/G2P[4] | HQ657167.1 |
| RVA/Human/IND/NR1/XXXX/GXP[X] | AF506018.1 |  | RVA/Human-tc/SWE/1076/1983/G2P2A[6] | JX416215.1 |
| RVA/Human-wt/AUS/336190/2004/G2P[4] | KC834694.1 |  | RVA/Human/KEN/D205/1989/G2P[4] | JF304922.1 |
| RVA/Human-wt/AUS/SA066/2010/G2P[4] | KC571498.1 |  | RVA/Goat-tc/BGD/GO34/1999/G6P[1] | GU937884.1 |
| RVA/Human-wt/BGD/MMC88/2005/G2P[4] | HQ641369.1 |  | RVA/Bovine/IND/UKD/IVRI/09/M-1/2009/G3P[11] | HM363561.1 |
| RVA/Human/RUS/Nov04-H391/2004/G2P[4] | KC020020.1 |  | RVA/Sheep-tc/ESP/OVR762/2002/G8P[14] | EF554155.1 |
| RVA/Human-wt/BGD/MMC6/2005/G2P[4] | HQ641360.1 |  | RVA/Human-wt/HUN/Hun5/1997/G6P[14] | EF554111.1 |
| RVA/Human-wt/AUS/V233/1999/G2P[4] | KC834695.1 |  | RVA/Human-tc/IND/69M/1980/G8P[10] | EF672559.1 |
| RVA/Human-wt/ZAF/GR10924/1999/G9P[6] | FJ183361.1 |  | RVA/Human-wt/CHN/TB-Chen/1996/G2P[4] | AY787648.1 |
| RVA/Human-wt/BGD/RV161/2000/G12P[6] | DQ490541.1 |  | RVA/Pidgeon-tc/JPN/PO-13/1983/G18P[17] | AB009625.2 |
| RVA/Human-wt/MWI/1473/2001/G8P[4] | HQ657134.1 |  |  |  |

Table S2. Continued

| **I. NSP3** | | | | |
| --- | --- | --- | --- | --- |
| Strain | Accession Number |  | Strain | Accession Number |
| RVA/Human-wt/HUN/BP1062/2004/G8P[14] | FN665695.1 |  | RVA/Human-wt/IND/mcs63/2011/G8P[4] | JX307623.1 |
| RVA/Human-wt/HUN/Hun5/1997/G6P[14] | EF554112.1 |  | RVA/Human-wt/AUS/V233/1999/G2P[4] | KC834692.1 |
| RVA/Goat-tc/BGD/GO34/1999/G6P[1] | GU937885.1 |  | RVA/Human-wt/THA/CU497-BK/09/2009/G2P[4] | JN706627.1 |
| RVA/Human-wt/BGD/Dhaka16/2003/G1P[8] | DQ492677.1 |  | RVA/Human-wt/ZAF/3203WC/2009/G2P[4] | HQ657168.1 |
| RVA/Human-wt/BEL/B3458/2003/G9P[8] | EF672558.1 |  | RVA/Human-wt/BEL/F01322/2009/G3P[6] | JF460831.1 |
| RVA/Human-wt/BEL/B4633/2003/G12P[8] | DQ146646.1 |  | RVA/Human-wt/BEL/F01498/2009/G3P[6] | JF460842.1 |
| RVA/Human-tc/GBR/ST3/1975/G4P2A[6] | EF672614.1 |  | RVA/Human-wt/ITA/CEC06/2011/G6P[6] | KC152913.1 |
| RVA/Human-wt/COD/DRC86/2003/G8P[6] | DQ005117.1 |  | RVA/Human-wt/USA/06-242/2006/G2P[6] | JF460820.1 |
| RVA/Human-wt/COD/DRC88/2003/G8P[8] | DQ005106.1 |  | RVA/Human-wt/ZAF/2371WC/2008/G9P[8] | JN013979.1 |
| RVA/Human-wt/KEN/MRC-DPRU1606/2009/G8P[4] | KF636242.1 |  | RVA/Human-wt/ITA/PA84/2008/G2P[4] | KC178743.1 |
| RVA/Human-wt/ZMB/MRC-DPRU3463/2009/G8P[4] | KF636308.1 |  | RVA/Human-wt/ZAF/MRC-DPRU1061/2009/G2P[4] | KF636319.1 |
| RVA/Human-wt/ZAF/GR10924/1999/G9P[6] | FJ183359.1 |  | RVA/Human-wt/AUS/RCH041/2010/G2P[4] | JX965152.1 |
| RVA/Human-wt/MWI/1473/2001/G8P[4] | HQ657135.1 |  | RVA/Human-wt/CHN/TB-Chen/1996/G2P[4] | AY787649.1 |
| RVA/Human-wt/BEL/B1711/2002/G6P[6] | EF554090.1 |  | RVA/Human-tc/KEN/D205/1989/G2P[4] | JF304923.1 |
| RVA/Human-wt/BGD/RV161/2000/G12P[6] | DQ490542.1 |  | RVA/Human-tc/IND/69M/1980/G8P[4][10] | EF672558.1 |
| RVA/Human-wt/BGD/RV176/2000/G12P[6] | DQ490559.1 |  | RVA/Sheep-tc/ESP/OVR762/2002/G8P[14] | EF554156.1 |
| RVA/Human-wt/BGD/MMC6/2005/G2P[4] | HQ641361.1 |  | RVA/Cow-tc/VEN/BRV033/1990/G6P[1] | KF636308.1 |
| RVA/Human-wt/BGD/MMC88/2005/G2P[4] | HQ641370.1 |  | RVA/Pidgeon-tc/JPN/PO-13/1983/G18P[17] | AB009626.2 |
| **J. NSP4** | | | | |
| Strain | Accession Number |  | Strain | Accession Number |
| RVA/Human-wt/COD/DRC86/2003/G8P[6] | DQ005116.1 |  | RVA/Cow-wt/ZAF/1605/2007/G6P[5] | JN831229.1 |
| RVA/Human-wt/COD/DRC88/2003/G8P[8] | DQ005105.1 |  | RVA/Human-wt/TUN/17237/2008/G6P[9] | JX271010.1 |
| RVA/Cow-wt/ZAF/1604/2007/G8P[1] | JN831218.1 |  | RVA/Human-tc/ITA/PA169/1988/G6P[14] | EF554135.1 |
| RVA/Antelope-wt/ZAF/RC-18-08/2008/G6P[14] | FJ495135.1 |  | RVA/Human-wt/BEL/B10925/1997/G6P[14] | EF554124.1 |
| RVA/Human-wt/BEL/B1711/2002/G6P[6] | EF554091.1 |  | RVA/Human-wt/ITA/111-05-27/2005/G6P[14] | EF554146.1 |
| RVA/Human-wt/ZAF/GR10924/1999/G9P[6] | FJ183363.1 |  | RVA/Sheep-tc/ESP/OVR762/2002/G8P[14] | EF554157.1 |
| RVA/Human-wt/CHN/TB-Chen/1996/G2P[4] | AY787650.1 |  | RVA/Human-wt/HUN/BP1062/2004/G8P[14] | FN665697.1 |
| RVA/Human-wt/BEL/F01322/2009/G3P[6] | JF460832.1 |  | RVA/Human-wt/HUN/Hun5/1997/G6P[14] | EF554113.1 |
| RVA/Human-wt//USA/06-242/2006/G2P[6] | JF460821.1 |  | RVA/Human-wt/KEN/B12/1987/G8P[1] | HM627551.1 |
| RVA/Human-wt/BGD/Dhaka16/2003/G1P[8] | DQ492678.1 |  | RVA/Human-tc/IND/69M/1980/G8P[10] | EF672561.1 |
| RVA/Human-wt/BGD/RV161/2000/G12P[6] | DQ490543.1 |  | RVA/Human-tc/SWE/1076/1983/G2P[4] | U59105.1 |
| RVA/Human-wt/BEL/B4633/2003/G12P[8] | DQ146647.1 |  | RVA/Goat-tc/BGD/GO34/1999/G6P[1] | GU937886.1 |
| RVA/Human-wt/BEL/B3458/2003/G9P[8] | EF990712.1 |  | RVA/Lamb/CHN/LLR/1985/G10P[12] | JQ031148.1 |
| RVA/Human-tc/GBR/ST3/1975/G4P2A[6] | U59110.1 |  | RVA/Pidgeon-tc/JPN/PO-13/1983/G18P[17] | AB009627.1 |
| RVA/Cow-wt/ZAF/1603/2007/G6P[5] | JN831207.1 |  |  |  |

Table S2. Continued

| **I. NSP5** | | | | |
| --- | --- | --- | --- | --- |
| Strain | Accession Number |  | Strain | Accession Number |
| RVA/Human-wt/BEL/B3458/2003/G9P[8] | EF990713.1 |  | RVA/Vaccine/USA/RotaTeq-WI79-9/1992/G1P7[5] | GU565062.1 |
| RVA/Human-wt/BEL/B4633/2003/G12P[8] | DQ146648.1 |  | RVA/Vaccine/USA/RotaTeq-WI79-4/1992/G6P1A[8] | GU565051.1 |
| RVA/Human-wt/BGD/Dhaka16/2003/G1P[8] | DQ492679.1 |  | RVA/Vaccine/USA/RotaTeq-SC2-9/1992/G2P7[5] | GU565073.1 |
| RVA/Human-tc/GBR/ST3/1975/G4P2A[6] | EF672618.1 |  | RVA/Bovine-tc/JPN/NCDV/1971/G6P[1] | GU937876.1 |
| RVA/Human-wt/BEL/B1711/2002/G6P[6] | EF554092.1 |  | RVA/Bovine/KOR/KJ19-2/2004/G6P[7] | FJ206054.1 |
| RVA/Human-wt/USA/06-242/2006/G2P[6] | JF460822.1 |  | RVA/Rhesus-tc/USA/PTRV/1990/G8P[1] | FJ422141.1 |
| RVA/Human-wt/CHN/TB-Chen/1996/G2P[4] | AY787651.1 |  | RVA/Antelope-wt/ZAF/RC-18-08/2008/G6P[14] | FJ495136.1 |
| RVA/Human-wt/ZAF/GR10924/1999/G9P[6] | FJ183362.1 |  | RVA/Human-wt/TUN/17237/2008/G6P[9] | JX271011.1 |
| RVA/Human-wt/BGD/RV161/2000/G12P[6] | DQ490544.1 |  | RVA/Human-wt/ITA/PAI58/1996/G3P[9] | GU296419.1 |
| RVA/Human-wt/BEL/F01322/2009/G3P[6] | JF460833.1 |  | RVA/Human/RUS/O1180/2011/G3P[9] | KC020048.1 |
| RVA/Human-wt/COD/DRC86/2003/G8P[6] | DQ005115.1 |  | RVA/Cow-wt/ARG/B383/1998/G15P[11] | FJ347121.1 |
| RVA/Human-wt/COD/DRC88/2003/G8P[8] | DQ005104.1 |  | RVA/Human-tc/JPN/AU-1/1982/G3P[9] | AB008656.1 |
| RVA/Human-tc/IND/69M/1980/G8P[10] | EF672562.1 |  | RVA/Lamb/CHN/CC0812-1/2008/G10P15 | HQ834206.1 |
| RVA/Human-tc/AUS/MG6/1993/G6P[14] | EF554103.1 |  | RVA/Goat-tc/BGD/GO34/1999/G6P[1] | GU937887.1 |
| RVA/Human-wt/AUS/RCH272/2012/G3P[14] | KF690135.1 |  | RVA/Cow-wt/ZAF/1603/2007/G6P[5] | JN831208.1 |
| RVA/Human-wt/BEL/B10925/1997/G6P[14] | EF554125.1 |  | RVA/Cow-wt/ZAF/1605/2007/G6P[5] | JN831230.1 |
| RVA/Sheep-tc/ESP/OVR762/2002/G8P[14] | EF554158.1 |  | RVA/Cow-wt/ZAF/1604/2007/G8P[1] | JN831219.1 |
| RVA/Human-wt/ITA/111-05-27/2005/G6P[14] | EF554147.1 |  | RVA/Guanaco-wt/ARG/Chubut/1999/G8P[14] | FJ347110.1 |
| RVA/Human-tc/KEN/B12/1987/G8P[1] | HM627552.1 |  | RVA/Human-wt/HUN/Hun5/1997/G6P[14] | EF554114.1 |
| RVA/Human-tc/ITA/PA169/1988/G6P[14] | EF554136.1 |  | RVA/Pidgeon-tc/JPN/PO-13/1983/G18P[17] | AB009628.1 |
