# Supplementary material for: Identification of Novel Ghanaian G8P[6] Human-Bovine Reassortant Rotavirus Strain by Next Generation Sequencing
Source: PLoS One. 2014 Jun 27;9(6):e100699. doi: 10.1371/journal.pone.0100699 (PMC4074113; doi:10.1371/journal.pone.0100699)
Supplement: Table S3 — Nucleotide sequence identities (%) of the full-length ORFs of all 11 gene segments of GH018-08 to selected human and animal reference rotavirus strains. (DOCX) [file pone.0100699.s004.docx]

Table S3. Nucleotide sequence identities (%) of the full-length ORFs of all 11 gene segments of GH018-08 to selected human and animal reference rotavirus strains

| Strain^a^ | Genotype^b^ (Nucleotide sequence identity (%) of full-length ORFs to RVA/Human-wt/GHA/GH018-08/2008/G8P[6]) | | | | | | | | | | | | | | | | | | | | | | | | | | | | | | | | | | | | | |  |  |
| --- | --- | --- | --- | --- | --- | --- | --- | --- | --- | --- | --- | --- | --- | --- | --- | --- | --- | --- | --- | --- | --- | --- | --- | --- | --- | --- | --- | --- | --- | --- | --- | --- | --- | --- | --- | --- | --- | --- | --- | --- |
|  | VP7 | | VP4 | | VP6 | VP1 | VP2 | | VP3 | |  | NSP1 | | |  | | NSP2 | |  | NSP3 | | | |  | NSP4 | | | | |  | NSP5 | | | | | | |  |  |  |
|  |  | |  | |  |  |  | |  | |  |  | | |  | |  | |  |  | | | |  |  | | | | |  |  | | | | | | |  |  |  |
| **RVA/Human-wt/GHA/GH018-08/2008/G8P[6]** | G8 (100) | | P[6] (100) | | I2 (100) | R2 (100) | C2 (100) | | M2 (100) | |  | A2 (100) | | |  | | N2 (100) | |  | T2 (100) | | | |  | E2 (100) | | | | |  | H3 (100) | | | | | | |  |  |  |
| **RVA/Human-wt/GHA/GH019-08/2008/G8P[6]** | **G8 (100)** | | **P[6] (100)** | | **I2 (100)** | **R2 (91.1)** | **C2 (99.6)** | | **M2 (99.9)** | |  | **A2 (100)** | | |  | | **N2 (100)** | |  | **T2 (99.9)** | | | |  | **E2 (100)** | | | | |  | **H3 (100)** | | | | | | |  |  |  |
|  |  | |  | |  |  |  | |  | |  |  | | |  | |  | |  |  | | | |  |  | | | | |  |  | | | | | | |  |  |  |
| RVA/Human-wt/CHN/TB-Chen/1996/G2P[4] | G2 (71.7) | | P[4] (74.3) | | **I2 (86.4)** | **R2 (90.2)** | **C2 (84.7)** | | **M2 (82.8)** | |  | **A2 (97.4)** | | |  | | **N2 (87.5)** | |  | **T2 (97.3)** | | | |  | **E2 (84.9)** | | | | |  | H2 (86.1) | | | | | | |  |  |  |
| RVA/Human-wt/USA/06-242/2006/G2P[6] | G2 (71.2) | | **P[6] (99.6)** | | **I2 (92.8)** | **R2 (91.0)** | **C2 (84.4)** | | **M2 (82.9)** | |  | **A2 (99.3)** | | |  | | **N2 (91.4)** | |  | **T2 (99.4)** | | | |  | **E2 (86.0)** | | | | |  | H2 (86.6) | | | | | | |  |  |  |
| RVA/Human-wt/BEL/F01322/2009/G3P[6] | G3 (74.9) | | **P[6] (97.7)** | | **I2 (92.6)** | **R2 (91.0)** | **C2 (84.5)** | | **M2 (82.7)** | |  | **A2 (99.1)** | | |  | | **N2 (91.3)** | |  | **T2 (99.1)** | | | |  | **E2 (85.8)** | | | | |  | H2 (86.4) | | | | | | |  |  |  |
| RVA/Human-wt/ZAF/GR10924/1999/G9P[6] | G9 (77.0) | | **P[6] (97.0)** | | **I2 (92.4)** | **R2 (91.6)** | **C2 (84.5)** | | **M2 (82.8)** | |  | **A2 (98.0)** | | |  | | **N2 (91.5)** | |  | **T2 (97.8)** | | | |  | **E2 (86.4)** | | | | |  | H2 (86.4) | | | | | | |  |  |  |
| RVA/Human-wt/BEL/B1711/2002/G6P[6] | G6 (77.0) | | **P[6] (97.4)** | | **I2 (92.9)** | **R2 (96.1)** | **C2 (84.5)** | | **M2 (86.7)** | |  | **A2 (97.2)** | | |  | | **N2 (91.5)** | |  | **T2 (97.3)** | | | |  | **E2 (86.0)** | | | | |  | H2 (86.4) | | | | | | |  |  |  |
| RVA/Human-wt/COD/DRC86/2003/G8P[6] | **G8 (96.7)** | | **P[6] (95.8)** | | **I2 (92.5)** | **R2 (91.0)** | **C2 (84.7)** | | **M2 (82.7)** | |  | **A2 (97.0)** | | |  | | **N2 (92.0)** | |  | **T2 (97.8)** | | | |  | **E2 (86.0)** | | | | |  | H2 (86.1) | | | | | | |  |  |  |
| RVA/Human-wt/COD/DRC88/2003/G8P[8] | **G8 (96.6)** | | P[8] (75.3) | | **I2 (92.5)** | **R2 (91.0)** | **C2 (84.5)** | | **M2 (82.8)** | |  | **A2 (97.1)** | | |  | | **N2 (91.9)** | |  | **T2 (97.4)** | | | |  | **E2 (85.6)** | | | | |  | H2 (86.1) | | | | | | |  |  |  |
| RVA/Human-tc/IND/69M/1980/G8P[10] | **G8 (84.5)** | | P[10] (71.5) | | **I2 (93.3)** | **R2 (86.6)** | **C2 (86.6)** | | **M2 (87.7)** | |  | **A2 (94.3)** | | |  | | **N2 (87.3)** | |  | **T2 (91.1)** | | | |  | **E2 (89.8)** | | | | |  | H2 (87.4) | | | | | | |  |  |  |
|  |  | |  | |  |  |  | |  | |  |  | | |  | |  | |  |  | | | |  |  | | | | |  |  | | | | | | |  |  |  |
| RVA/Human-wt/HUN/BP1062/2004/G8P[14] | **G8 (86.8)** | | P[14] (65.7) | | **I2 (95.1)** | **R2 (89.3)** | **C2 (87.7)** | | **M2 (83.6)** | |  | A11 (64.9) | | |  | | **N2 (92.7)** | |  | T6 (77.3) | | | |  | **E2 (93.9)** | | | | |  | **H3 (95.3)** | | | | | | |  |  |  |
| RVA/Sheep-tc/ESP/OVR762/2002/G8P[14] | **G8 (83.4)** | | P[14] (65.9) | | **I2 (93.7)** | **R2 (86.3)** | **C2 (87.0)** | | **M2 (82.2)** | |  | A11 (67.4) | | |  | | **N2 (87.7)** | |  | T6 (78.1) | | | |  | **E2 (93.8)** | | | | |  | **H3 (96.0)** | | | | | | |  |  |  |
| RVA/Human-wt/HUN/Hun5/1997/G6P[14] | G6 (75.9) | | P[14] (65.7) | | **I2 (88.4)** | **R2 (87.0)** | **C2 (86.5)** | | **M2 (82.9)** | |  | A11 (66.8) | | |  | | **N2 (88.1)** | |  | T6 (78.2) | | | |  | **E2 (93.8)** | | | | |  | **H3 (95.3)** | | | | | | |  |  |  |
| RVA/Goat-tc/BGD/GO34/1999/G6P[1] | G6 (77.4) | | P[1] (70.6) | | **I2 (92.3)** | **R2 (91.8)** | **C2 (89.0)** | | **M2 (83.5)** | |  | A11 (67.4) | | |  | | **N2 (91.8)** | |  | T6 (78.0) | | | |  | **E2 (87.9)** | | | | |  | **H3 (95.5)** | | | | | | |  |  |  |
|  |  | |  | |  |  |  | |  | |  |  | | |  | |  | | | |  |  | | | |  |  | | | | |  |  | | | | | | |  |
| RVA/Human-wt/BGD/Dhaka16/2003/G1P[8] | G1 (72.2) | | P[8] (75.0) | | I1 (80.6) | R1 (78.6) | C1 (80.2) | | M1 (76.3) | |  | A1 (75.4) | | |  | | N1 (82.6) | |  | T1 (78.4) | | | |  | E1 (79.2) | | | | |  | H1 (88.6) | | | | | | |  |  |  |
| RVA/Human-tc/GBR/ST3/1975/G4P2A[6] | G4 (71.4) | | **P[6] (95.1)** | | I1 (80.1) | R1 (79.3) | C1 (80.2) | | M1 (76.4) | |  | A1 (75.0)* | | |  | | N1 (82.6) | |  | T1 (78.7) | | | |  | E1 (79.0) | | | | |  | H1 (88.4) | | | | | | |  |  |  |
| RVA/Human-wt/BEL/B3458/2003/G9P[8] | G9 (76.7) | | P[8] (74.9) | | I1 (79.8) | R1 (78.4) | C1 (80.1) | | M1 (76.4) | |  | A1 (75.5) | | |  | | N1 (85.2) | |  | T1 (78.4) | | | |  | E1 (79.6) | | | | |  | H1 (88.2) | | | | | | |  |  |  |
| RVA/Human-wt/BEL/B4633/2003/G12P[8] | G12 (73.1) | | P[8] (75.0) | | I1 (80.4) | R1 (78.7) | C1 (80.0) | | M1 (76.2) | |  | A1 (75.2) | | |  | | N1 (82.8) | |  | T1 (78.1) | | | |  | E1 (79.9) | | | | |  | H1 (88.6) | | | | | | |  |  |  |
|  | |  | |  | |  | |  | |  | | |  |  | |  | |  | | | | |  | | | | |  |  | | | | |  |  |  |  | | | |

^a^Names of RVA strains sequenced in this study are shown in **boldface** font; animal strains are indicated by blue font.

^b^Gene segments with a genotype identical to RVA/Human-wt/GHA/GH018-08/2008/G8P[6] are indicated by **boldface** font; green box: Wa-like gene segments; red box: DS-1-like gene segments; orange box: AU-1-like gene segments; purple box: typical bovine/artiodactyl-like gene segments; blue box: G8 strains. Strains with highest nucleotide identities are indicated by red font.

*Complete ORF of RVA/Human-tc/GBR/ST3/1975/G4P2A[6] is relatively shorter [1461 nt, 486 aa).

VP1 gene nucleotide sequence identity (%) of GH018-08 to GH019-08 is highlighted in yellow

GenBank accession numbers for reference strains are listed in Table S2.:
