# Supplementary material for: Identification of Novel Ghanaian G8P[6] Human-Bovine Reassortant Rotavirus Strain by Next Generation Sequencing
Source: PLoS One. 2014 Jun 27;9(6):e100699. doi: 10.1371/journal.pone.0100699 (PMC4074113; doi:10.1371/journal.pone.0100699)
Supplement: Table S4 — Nucleotide sequence identities (%) of the full-length ORFs of all 11 gene segments of GH018-08 to selected, relevant ‘non-reference’ human and animal rotavirus strains. (DOCX) [file pone.0100699.s005.docx]

Table S4. Nucleotide sequence identities (%) of the full-length ORFs of all 11 gene segments of GH018-08 to selected, relevant ‘non-reference’ human and animal rotavirus strains

| Strain^a^ | Genotype^b^ (Nucleotide sequence identity (%) of full-length ORFs to RVA/Human-wt/GHA/GH018-08/2008/G8P[6]) | | | | | | | | | | | | | | | |
| --- | --- | --- | --- | --- | --- | --- | --- | --- | --- | --- | --- | --- | --- | --- | --- | --- |
|  | VP7 | VP4 | VP6 | VP1 | VP2 | VP3 |  | NSP1 |  | NSP2 |  | NSP3 |  | NSP4 |  | NSP5 |
|  |  |  |  |  |  |  |  |  |  |  |  |  |  |  |  |  |
| RVA/Human/1290/Kenya/1991/G8P[X] | **G8 (97.7)** | - | - | - | - | - |  | - |  | - |  | - |  | - |  | - |
| RVA/Simian/KEN/KY1646/1999/G8P[6] | **G8 (97.3)** | - | - | - | - | - |  | - |  | - |  | - |  | - |  | - |
| RVA/Human/NGR/HMG89/XXXX/G8P[X] | **G8 (97.2)** | - | - | - | - | - |  | - |  | - |  | - |  | - |  | - |
| RVA/Human-tc/MWI/MW333/1997/G8P[4] | **G8 (96.8)** | - | - | - | - | - |  | - |  | - |  | - |  | - |  | - |
| RVA/Human-tc/MWI/MW23/1997/G8P[6] | **G8 (96.7)** | **P[6] (96.5)** | - | - | - | - |  | - |  | - |  | - |  | - |  | - |
| RVA/Bovine-tc/NGR/NGRBg8/1998/G8P[1] | **G8 (96.0)** | - | - | - | - | - |  | - |  | - |  | - |  | - |  | - |
| RVA/Human-tc/KEN/B12/1987/G8P[1] | **G8 (84.4)** | - | - | - | **C2 (87.0)** | **M2 (88.6)** |  | - |  | - |  | - |  | **E2 (91.1)** |  | **H3 (96.0)** |
| RVA/Cow-wt/IND/970/2009/G3P[X] | - | - | **I2 (97.7)** | - | - | - |  | - |  | - |  | - |  | - |  | - |
| RVA/Bovine-wt/IND/UKD/P14/2009/G3P[1] | - | - | **I2 (97.3)** | - | - | - |  | - |  | - |  | - |  | - |  | - |
| RVA/Porcine-wt/IND/HP140/XXXX/G6P[13] | - | - | **I2 (98.1)** | - | - | - |  | - |  | - |  | - |  | - |  | - |
| RVA/Porcine-wt/IND/HP113/XXXX/G6P[13] | - | - | **I2 (98.8)** | - | - | - |  | - |  | - |  | - |  | - |  | - |
| RVA/Camel-wt/SDN/MRC-DPRU447/2002/G8P[11] | - | - | - | **R2 (90.8)** | - | **M2 (88.9)** |  | - |  | - |  | - |  | - |  | - |
| RVA/Cow/IND/M1/UKD/2010/GXP[X] | - | - | - | - | **C2 (97.2)** | - |  | - |  | - |  | - |  | - |  | - |
| RVA/Bovine-tc/ZAF/'O'Agent/1965/G8P[1] | - | - | - | - | **C2 (87.1)** | **M2 (90.1)** |  | - |  | - |  | - |  | - |  | - |
| RVA/Caprine/CHN/XL/2010/G10P[15] | - | - | - | - | **C2 (89.2)** | - |  | - |  | - |  | - |  | - |  | - |
| RVA/Lamb/CHN/CC0812-1/2008/G10P[15] | - | - | - | - | **C2 (89.3)** | - |  | - |  | **N2 (90.4)** |  | - |  | - |  | **H3 (94.8)** |
| RVA/Lamb/CHN/LLR/1985/G10P[12] | - | - | - | - | **C2 (89.3)** | - |  | - |  | - |  | - |  | E2 (86.2) |  | - |
| RVA/Lamb/CHN/Lamb-NT/2007/G10P[15] | - | - | - | - | **C2 (89.1)** | - |  | - |  | **N2 (90.3)** |  | - |  | - |  | - |
| RVA/Human-tc/KEN/D205/1989/G2P[4] | - | - | - | - | - | **M2 (88.8)** |  | **A2 (95.9)** |  | **N2 (92.4)** |  | **T2 (96.4)** |  | - |  | - |
| RVA/Human-wt/ZAF/MRC-DPRU9317/1999/G9P[6] | - | - | - | - | - | - |  | **A2 (98.0)** |  | - |  | - |  | - |  | - |
| RVA/Horse-wt/ZAF/EqRV-SA1/2006/G14P[12] | - | - | - | - | **C2 (86.6)**- | - |  | - |  | **N2 (91.5)** |  | - |  | - |  | - |
| RVA/Horse-wt/IRL/04V2024/2004/G14P[12] | - | - | - | - | **C2 (86.5)** | - |  | - |  | **N2 (91.7)** |  | - |  | - |  | - |
| RVA/Human-wt/ZAF/2371WC/2008/G9P[8] |  |  |  |  |  |  |  |  |  |  |  | **T2 (99.4)** |  | - |  | - |
| RVA/Human-wt/BEL/B10925/1997/G6P[14] | - | - | - | - | - | - |  | - |  | - |  | - |  | **E2 (94.1)** |  | **H3 (96.3)** |
| RVA/Human-wt/ITA/111-05-27/2005/G6P[14] | - | - | - | - | - | - |  | - |  | - |  | - |  | **E2 (93.6)** |  | **H3 (96.5)** |
| RVA/Cow-wt/ZAF/1603/2007/G6P[5] | - | - | - | - | - | - |  | - |  | - |  | - |  | **E2 (90.3)** |  | **H3 (95.6)** |
| RVA/Cow-wt/ZAF/1605/2007/G6P[5] | - | - | - | - | - | - |  | - |  | - |  | - |  | **E2 (90.2)** |  | **H3 (95.6)** |
| RVA/Cow-wt/ZAF/1604/2007/G8P[1] | - | - | - | - | - | - |  | - |  | - |  | - |  | - |  | **H3 (95.6)** |
| FJ206054.1_RVA/Bovine/KOR/KJ19-2/2004/G6P[7] | - | - | - | - | - | - |  | - |  | - |  | - |  | - |  | **H3 (97.0)** |
| FJ347121.1_RVA/Cow-wt/ARG/B383/1998G15P[11] |  |  |  |  |  |  |  |  |  |  |  |  |  |  |  | **H3 (96.7)** |
|  |  |  |  |  |  |  |  |  |  |  |  |  |  |  |  |  |

^a^Names of animal strains are indicated by blue font.

^b^Gene segments of non-reference strains with a genotype identical to RVA/Human-wt/GHA/GH018-08/2008/G8P[6] are indicated by **boldface** font; red box: DS-1-like gene segments; orange box: AU-1-like gene segments. Strains with highest nucleotide identities are indicated by red font

GenBank accession numbers for strains are listed in Table S2
